# Regression discontinuity design for the study of health effects of exposures acting early in life

**DOI:** 10.3389/fpubh.2024.1377456

**Published:** 2024-04-19

**Authors:** Maja Popovic, Daniela Zugna, Kate Tilling, Lorenzo Richiardi

**Affiliations:** ^1^Cancer Epidemiology Unit, Department of Medical Sciences, University of Turin and CPO-Piemonte, Turin, Italy; ^2^MRC Integrative Epidemiology Unit at the University of Bristol, University of Bristol, Bristol, United Kingdom; ^3^Population Health Sciences, Bristol Medical School, University of Bristol, Bristol, United Kingdom

**Keywords:** regression discontinuity, epidemiology, review, DOHaD, RDD, early life exposures, health effects

## Abstract

Regression discontinuity design (RDD) is a quasi-experimental approach to study the causal effect of an exposure on later outcomes by exploiting the discontinuity in the exposure probability at an assignment variable cut-off. With the intent of facilitating the use of RDD in the Developmental Origins of Health and Disease (DOHaD) research, we describe the main aspects of the study design and review the studies, assignment variables and exposures that have been investigated to identify short- and long-term health effects of early life exposures. We also provide a brief overview of some of the methodological considerations for the RDD identification using an example of a DOHaD study. An increasing number of studies investigating the effects of early life environmental stressors on health outcomes use RDD, mostly in the context of education, social and welfare policies, healthcare organization and insurance, and clinical management. Age and calendar time are the mostly used assignment variables to study the effects of various early life policies and programs, shock events and guidelines. Maternal and newborn characteristics, such as age, birth weight and gestational age are frequently used assignment variables to study the effects of the type of neonatal care, health insurance, and newborn benefits, while socioeconomic measures have been used to study the effects of social and welfare programs. RDD has advantages, including intuitive interpretation, and transparent and simple graphical representation. It provides valid causal estimates if the assumptions, relatively weak compared to other non-experimental study designs, are met. Its use to study health effects of exposures acting early in life has been limited to studies based on registries and administrative databases, while birth cohort data has not been exploited so far using this design. Local causal effect around the cut-off, difficulty in reaching high statistical power compared to other study designs, and the rarity of settings outside of policy and program evaluations hamper the widespread use of RDD in the DOHaD research. Still, the assignment variables’ cut-offs for exposures applied in previous studies can be used, if appropriate, in other settings and with additional outcomes to address different research questions.

## 1 Introduction

The Developmental Origins of Health and Disease (DOHaD) has been consolidated as a concept asserting the causal effects of early life environmental stressors on health outcomes and identifying critical windows for prevention of later diseases (1). While early DOHaD research was mostly based on administrative data and registries, an increasing number of birth cohort studies have provided extensive datasets, covering information across multiple life stages, for current and future studies. Even with a growing number of data sources and the widespread availability of data, the main methodological challenges in DOHaD research and life course epidemiology remain the selection of appropriate study design for complex research questions, managing multiple relationships of biological and contextual variables, dealing with repeated measures over time, and, especially, controlling for confounders to mitigate residual confounding (2).

The issue of uncontrolled confounding, i.e., the violation of the exchangeability assumption, is probably the main obstacle to causal inference within the context of non-experimental studies, and several analytical and design approaches have been developed to control for confounding and obtain a potentially unbiased estimate of the exposure effect. Here, we focus on regression discontinuity design (RDD), a quasi-experimental approach that has been widely applied in the context of natural experiments (3, 4), and is becoming more common also in DOHaD and lifecourse epidemiology literature (4–6). With the intent of facilitating the use of RDD in these contexts, we describe the main aspects of the study design and review the studies, assignment variables and exposures that have been used to identify health effects of early life exposures. Finally, we provide a brief overview of some of the methodological considerations for the RDD identification and the design validity checks using an example of a study on health effects of an early life exposure.

## 2 Regression discontinuity design – basic concepts and frameworks

RDD, introduced in the 1960s, Thistlethwaite and Campbell (7) is a quasi-experimental design that shares similarities with randomized controlled trials, but lacks the completely random assignment to the intervention (intervention, treatment, or exposure, hereafter referred to as exposure in general). It typically implies that whoever imposes a certain policy, program, or clinical decision, controls the assignment to the exposure using an *a priori* decided criterion (e.g., an eligibility rule, or a clinical decision-making guideline). The exposure assignment in RDD studies is thus based on the cut-off value of an assignment variable (also referred to in the literature as the “forcing,” “rating” or the “running” variable) that creates a discontinuity in the probability of the exposure at the cut-off point. The assignment variable can be any continuous or discrete variable that individuals cannot manipulate to systematically place themselves above or below the cut-off. The stronger is the individuals’ inability to control their own value of the assignment variable the more valid is the design to identify the causal effects. The exogeneity of the cut-off value in the assignment variable implies that individuals just below the cut-off are on average similar in all observed and unobserved baseline characteristics to those just above the cut-off except for the exposure of interest, i.e., they are exchangeable. The exposure groups are only exchangeable very close to the cut-off, rendering the validity of the design plausible for relatively narrow windows around the assignment variable cut-off. If the assumption of exchangeability holds, any difference in the outcome (or in its probability function in the case of binary outcomes) on the two sides of the assignment variable cut-off will be caused by the exposure. The magnitude of the discontinuity in the outcome at the cut-off represents thus the average effect of the assignment rule around the cut-off point.

In summary, the RDD draws on a continuous or discrete pre-exposure variable with a clearly defined cut-off value for the exposure assignment that cannot be manipulated by the individuals. The cut-off refers to a specific exposure that can be studied in relationship with multiple outcomes. This is appealing for DOHaD research as many exposures acting at critical time windows early in life often have multiple short- and long-term health effects, which offers the opportunity to study different research questions using the same RDD setting.

**Figure 1:**
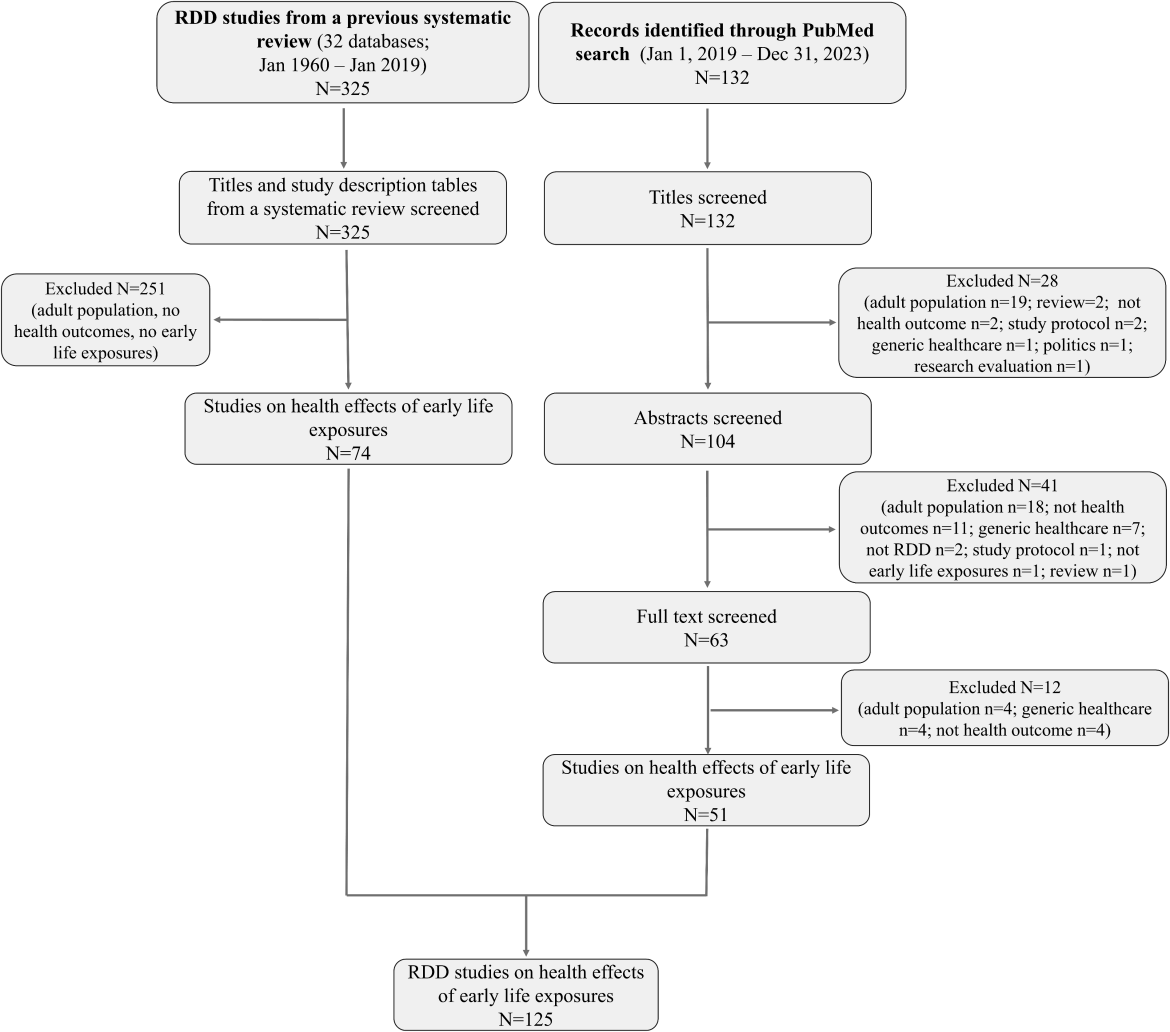
Flow diagram for the selection of RDD studies on the health effects of exposures acting early in life.

There are two main conceptual and inference frameworks in RDD: the continuity-based approach, and the local randomization approach (8–11). The continuity-based framework assumes the continuity of average potential outcomes near the cut-off, and it typically uses polynomial methods to approximate the regression functions on the two sides of the cut-off (polynomial of the observed outcome on the assignment variable) (9, 10, 12–14). In other words, it aims at estimating the difference between the two average potential outcomes at the cutoff; since this difference cannot be observed, it assumes that the average potential outcomes change continuously and in parallel around the cutoff. According to the local randomization framework, instead, RDD is seen as a randomized experiment near the cut-off, which assumes random assignment in a narrow window around the cut-off with the assignment variable being unrelated to the average potential outcomes (11, 15–17). It is thus assumed perfect exchangeability within the narrow window, with the aim of estimating the difference between the two average potential outcomes in the narrow window. The difference between the two frameworks is well explained for example in a recent tutorial by Cattaneo et al. (18), which Figure 1 provides a clear graphical representation of the assumed behavior of the average potential outcomes in the two frameworks. Local randomization imposes stronger assumptions than the continuity framework and is generally used when the sample size around the cutoff is small or in cases where the continuity framework cannot be applied because the assignment variable is discrete.

**Figure 2:**
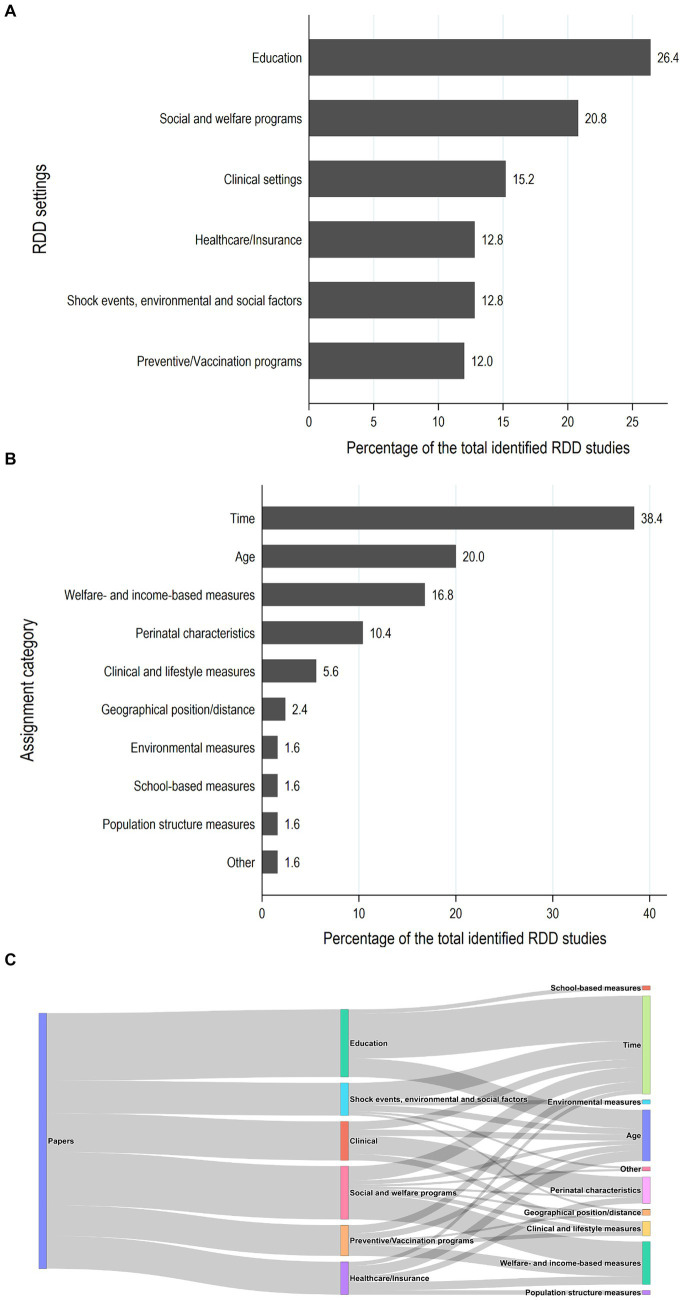
Summary of the settings **(A)** and assignment variables **(B)** used in RDD studies on health effects of early life exposures (*N* = 125). The Sankey diagram in **(C)** depicts the connections between assignment variables and the study settings in which they were used.

## 3 RDD applications for the study of health effects of exposures acting early in life

### 3.1 Studies

Three review articles have evaluated the application of RDD in health research (4–6), and one recent tutorial provided a guidance to RDD analysis with empirical examples from medical research (18). The most recent and the only systematic review (4), that performed searches of articles published until 2019 in several economic, social, and medical databases, identified 325 studies using RDD in the context of health research. The authors showed an increasing popularity of this design with most studies being applied in the context of specific policies, social programs, health insurance, and education. The review summarizes the mostly used assignment variables (e.g., age, date, socio-economic, clinical, and environmental measures) and the cut-off rules (program eligibility, legislation cut-offs, date of sudden events, and clinical decision-making rules) (4).

From the systematic review (4) and by updating the search until January 1, 2024, we identified RDD studies on health effects of exposures acting in fetal life, infancy, childhood, or adolescence with the aim of understanding the potential of promoting the use of RDD in DOHaD research. Identification of studies, search strategy, and the selected studies are detailed in Supplementary material S1 (Supplementary Methods, Identification of studies focused on health effects of exposures acting early in life, Supplementary Tables S1–S3). Overall, we identified 125 RDD studies on health effects of early life exposures (Figure 2).

Figure 2 shows the distribution of settings and assignment variables used in the identified RDD studies. Sixty percent of the identified studies (75/125) were conducted in the context of education (mostly based on educational reforms on schooling initiation and duration), social and welfare programs and policies (e.g., conditional cash transfers, child supplements, and parental leaves), and healthcare organization and insurance schemes. About 15% of the studies evaluated research questions related to clinical settings and patient care (*N* = 19). Interestingly, almost one third of the RDD studies on health effects of early life exposures published since 2019 focused on this setting, which was quite neglected in the past publications (6.8% of the studies published until 2019 and 27.5% of the studies published thereafter, Supplementary Figure S1). The number of RDD studies based on shock events, social and environmental factors has also increased substantially since 2019 (Supplementary Figure S1), probably also due to the increasing number of studies focused on the recent COVID-19 pandemic (19–21).

Almost 60% of the RDD studies on health effects of early life exposures used time and/or age as an assignment variable (Figure 2B). These assignment variables were also applied in almost all the studies conducted within the educational setting and settings based on shock events, social and environmental factors (Figure 2C). Frequently used assignment variables in previous studies also include welfare- and income-based individual and population measures and perinatal characteristics, mostly birthweight and gestational age. The latter were largely used within the clinical obstetrician/neonatal management and healthcare insurance settings.

As PubMed is the most extensively used database and search engine in DOHaD research, we described, among studies identified from the previous systematic review (4), the proportion of those present in PubMed. More than a half of the studies in each of the settings, except the clinical setting, are not available in PubMed (Supplementary Figure S1). Similarly, some of the assignment variables, such as geographical position or distance, environmental, population structure, and school-based measures were exclusively used in studies not available through a PubMed search (Supplementary Figure S2). Many of these articles, although with the focus on health effects of early life exposures, do not get the attention of DOHaD researchers.

Most of the identified RDD studies were setting-specific evaluating the effect of specific programs, policies, and sudden events, and are, thus, difficult to implement or replicate in different contexts and populations. However, there are some previous applications that used data that are typically collected in population registries and birth cohorts and may serve as motivating examples for future studies. Table 1 summarizes some of the assignment variables used for identification of discontinuities in studies on health effects of early life exposures that could be replicated or extended for future DOHaD research.

**Table 1:** Assignment variables and exposures as possible RDD models for DOHaD research.

Assignment variable	Determined cut-off values	Possible exposures / interventions
Birth weight	Low-birth weight (<2,500 grams) Very low birth weight (<1,500 grams) Extremely low birth weight (<1,000 grams) High birth weight (>4,000 or > 5,000 grams)	Extra neonatal care Neonatal intensive care unit Rooming-in and mother–child bonding Breastfeeding Caesarean section Specific treatments (e.g., probiotic supplementation, surfactant therapy) Setting-specific health insurance and welfare benefits
Gestational age	Preterm (<37 gestational weeks) Very preterm (<32 gestational weeks) Extremely preterm (<28 gestational weeks)	
Maternal age at conception	<18 years >35 years >40 years	Minimum cigarette/alcohol purchase age Screening and procedures for high-risk pregnancies
Socioeconomic measures	Setting-specific	Social, welfare, and cash transfer programs Health insurance policies
Parity	Setting-specific	
Age or date/year of birth	Setting-specific	Introduction of: Vaccination campaigns Pregnancy-specific guidelines Maternity/paternity leave policies Child-support grants Social and welfare programs
Calendar time	Setting-specific	Introduction of specific programs and policies Shock events
Clinical measures	Setting-specific	Treatment initiation Preventive programs
Levels of environmental measurements	Setting-specific	Local interventions (e.g., to reduce air-pollution)

Previous studies using the listed RDD settings are detailed and referenced in Supplementary material S1.

### 3.2 Methodological considerations

#### 3.2.1 Assignment rule condition

In RDD, the exposure is determined by the assignment rule either completely (deterministically) or partially (probabilistically). When the assignment rule perfectly determines the exposure (from 0 to 1 at the cut-off), the regression discontinuity takes a sharp design. This means that all individuals above the cut-off are assigned to an exposure and are exposed, while all those below the cut-off are assigned to the unexposed group, with no crossovers. If the assignment rule affects the probability of exposure creating a discontinuous change at the threshold, without an extreme 0 to 1 jump, regression discontinuity takes a fuzzy design. In this setting, there are exposed and unexposed individuals both above and below the cut-off, but the probability of being exposed jumps discontinuously at the cut-off (3, 8). Most of the applications of the RDD on the health effects of exposures acting early in life used a fuzzy design.

**Figure 3:**
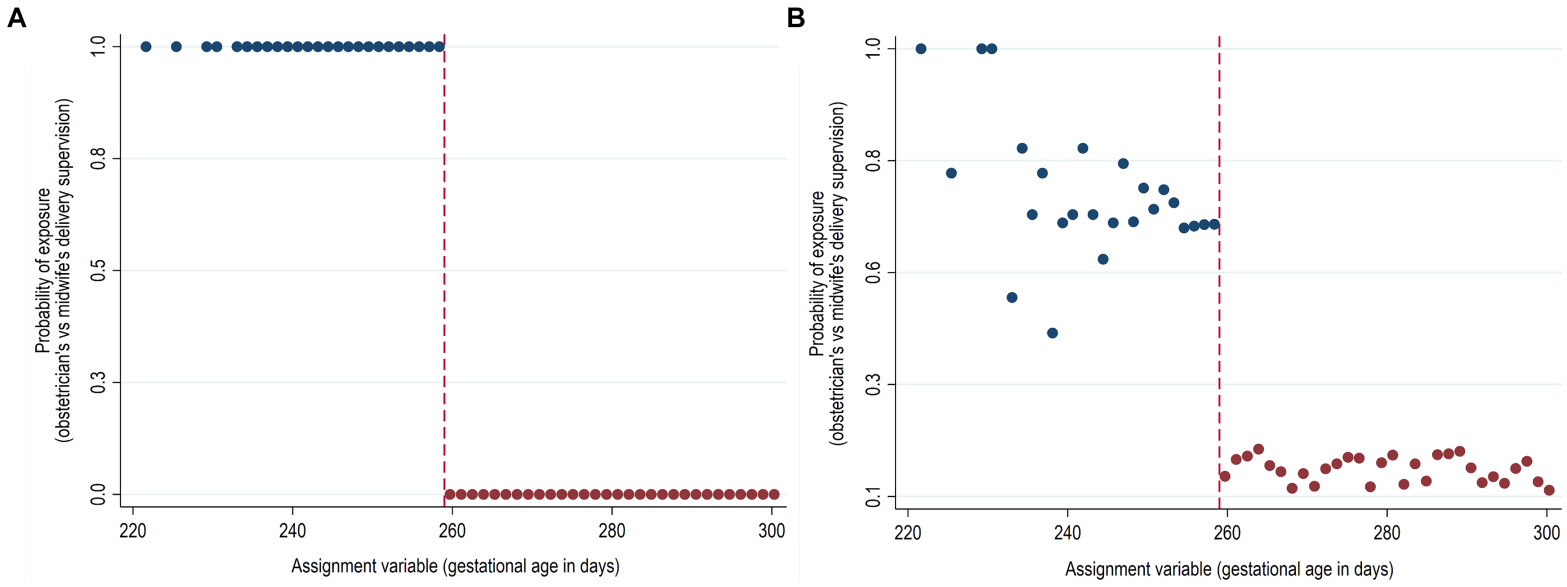
Hypothetical sharp and fuzzy regression discontinuity design. **(A)** Sharp (deterministic) regression discontinuity design. **(B)** Fuzzy (probabilistic) regression discontinuity design. Simulated data.

An example of the sharp and fuzzy RDD is illustrated in Figure 3 using simulated data motivated by the study of Daysal et al. (22). The study investigated the effect of the obstetrician supervision of deliveries on the short-term infant health outcomes using a national rule of 37 gestational weeks (259 days) at delivery for obstetrician instead of midwife delivery supervision. Data simulation is detailed in Supplementary material S1 (Methods). As shown in Figure 3A, a sharp design would imply that all deliveries before 37 gestational weeks were supervised by obstetricians and all those of at least 37 weeks were supervised by midwifes only. A fuzzy design, instead, would look like in Figure 3B in which the cut-off point decreases the probability of obstetrician supervision but does not completely determine it. This happens, for example when some deliveries after 37 gestational weeks are under the care of an obstetrician for reasons other than prematurity, such as complications during delivery and slow delivery progression.

Several modifications to the two general RDD settings have been proposed in literature, as for example the kink design where the assignment variable cut-off determines the change in the first derivative of the exposure probability (23), assignment variables with multiple cut-offs (24), RDD with multiple assignment variables (25, 26), or designs that exploit calendar time as the assignment variable (RDD in time) (27). The latter is an increasingly popular application of the RDD that uses time as the assignment variable, with an exposure date as the cut-off (Figure 1). RDD in time is closely related to other time-series designs, such as interrupted time series or pre-post analyses. The decision to use RDD in time compared to other time-series methods is particularly determined by the context and whether an exposure evolves over time, the type of collected data and the number of observations near the cut-off. If there are enough observations near the cut-off, an exposure does not change in time, and individual data are collected, the RDD assumptions may be highly plausible (27).

Whether the RDD has a sharp or a fuzzy design has implications on the assumptions summarized in Table 2. As the validity of RDD relies on these key underlying assumptions, Table 2 also summarizes their potential to be verified empirically. Using the simulated example described above, we briefly describe these assumptions and possible sensitivity analyses and falsification tests used to provide empirical evidence in case some of them are violated.

**Table 2:** The main assumptions of RDD.

Assumption	Description	Sharp RDD	Fuzzy RDD	Empirically verifiable
Relevance assumption	A continuous or discrete pre-exposure variable with a clearly defined cut-off value for the exposure assignment (the assignment rule causes exposure).	✓	✓	Yes
Exogeneity assumption and the lack of manipulation in the assignment variable	The cut-off is “as good as random” i.e., it is unrelated to the observed or unobserved factors.	✓	✓	Partially (observed factors and theoretical justification)
	The cut-off value is unrelated to individuals’ value of the assignment variable, and individuals’ assignment variable values are not determined by the cut-off of the assignment variable.	✓	✓	Yes
Exclusion restriction assumption	The assignment rule only affects the average outcomes through its effect on the exposure status, i.e., the same cut-off value is not used to assign the individuals to other exposures that influence the outcome.	✓	✓	Partially (observed factors and theoretical justification)
Continuity of the potential outcomes at the assignment variable cut-off (exchangeability)	Similarity of the individuals below and above the cut-off in potential outcomes and any unobserved factors	✓	✓	No
	Similarity of the individuals close to the cut-off in observed factors	✓	✓	Yes
	Outcome (or its probability function in the case of binary outcomes) is continuous at the cut-off in the absence of exposure	✓	✓	Yes
Local monotonicity assumption	The assignment rule must not increase the exposure in some people and decrease in others	⨯	✓	No

The listed assumptions refer mainly to the continuity-based methodological framework, while the local randomization framework requires additional, more stringent, assumptions.

#### 3.2.2 The main assumptions of RDD

##### 3.2.2.1 Relevance assumption

The assignment rule can be assessed empirically by plotting the relationship between the exposure and the assignment variable. Returning to the previous example illustrated in Figure 3, a discontinuous change in the probability of the obstetrician vs. midwife delivery supervision at the gestational age cut-off of 259 days suggests the possibility of using RDD, as the assignment variable at the cut-off should cause the exposure. For example, in all the identified RDD studies which used gestational age as the assignment variable for the obstetrician vs. midwife delivery supervision (37 weeks cut-off) (22, 28), antenatal corticosteroid administration (29, 30), or probiotic supplementation (34 weeks cut-off) (31) the relevance assumption was assessed by plotting and/or estimating the relationship between gestational age cut-off and the exposure of interest. The relevance assumption in RDD is analogous to the same assumption in an instrumental variable (IV) setting. As with an IV, the stronger the relationship between the instrument and the exposure variable, that is, the larger the discontinuity at the cut-off is, the more efficient and less prone to weak instrument bias the estimates from RDD are. While in sharp RDD the graphical representation is usually enough, fuzzy RDD with small discontinuities may require formal tests, such as F-statistics. As a rule of thumb, the instrument is considered weak if the F-statistic is less than 10 (32).

In addition to the exposure discontinuity at the cut-off, a graphical presentation of the relationship between the exposure and the assignment variable allows the examination of discontinuities in the exposure at locations other than the cut-off. While the absence of any such additional discontinuities is not a necessary condition to validate the RDD, their existence might indicate other exposures that could confound the estimate of the causal effect of the main exposure.

##### 3.2.2.2 Exogeneity assumption and the lack of manipulation in the assignment variable

The assignment variable should not only be a strong determinant of the exposure, but the cut-off value should also be exogenous and there should be no manipulation of the assignment variable by individuals. The exogeneity assumption implies that, conditional on the assignment variable, there are no other systematic differences between those below and above the assignment variable cut-off. In other words, any differences in outcomes between the exposure and control groups can be attributed solely to the exposure itself, rather than to any other confounding variables that might be correlated with both the assignment variable and the outcome. This assumption can be partly verified by checking whether the observed baseline characteristics have a similar distribution above and below the cut-off (see below). The exogeneity assumption also implies that the individuals cannot influence whether they are placed above or below the assignment variable cut-off. In practice, when there is a benefit in receiving an exposure, the manipulation in assignment variable occurs when the exposure assignment rule is public knowledge and individuals just barely qualifying for a desired exposure manage to cross the cut-off, with few individuals remaining on the other side of the cut-off. For instance, in a RDD studying the impact of a program that offers financial aid to students who score above a certain grade threshold on an exam, students might, by studying harder, attempt to manipulate their scores around the cut-off to ensure they qualify for the aid. Thus, it is crucial to have a deep understanding of the data generation process underlying the assignment rule. There is empirical evidence of manipulation when the distribution of the assignment variable shows a discontinuity at the cut-off. This can be checked visually using a density plot of the assignment variable as shown in Supplementary Figure S3 with our simulated example and applied in four out of five identified studies which used gestational age as the assignment variable (22, 28, 30, 31). Although previous studies found no evidence of manipulation near the 37 gestational week cut-off (22, 28), induction of labor for medical reasons at 37 weeks is not uncommon practice. Manipulation in the assignment variable at the cut-off can be formally tested using the McCrary density test (33), which tests the null hypothesis that the marginal density of the assignment variable is continuous around the cut-off.

##### 3.2.2.3 Exclusion restriction assumption

The exclusion restriction assumption is characteristic of the IV design, and it requires that the assignment rule affects the outcome exclusively through its effect on the exposure. The underlying assignment process in RDD must be known *a priori*, and alternative hypotheses must be excluded by providing evidence (often only theoretical) that the same assignment variable cut-off value is not used to assign the individuals to other exposures that could affect the outcome. In the context of studies assessing health effects of early life exposure this assumption may be violated if the same assignment variable cut-off, as for example widely used 2,500 grams birth weight cut-off for low birth weight babies or 37 gestational weeks cut-off for preterm birth, is used to determine several clinical decisions, like extra neonatal care, admission to neonatal intensive care unit, specific treatments or welfare benefits. If more than one of these exposures is likely to influence the outcome of interest it will not be possible to attribute the causal effect estimated using the RDD approach to a single exposure. Since the exclusion restriction assumption is untestable, it is important to provide theoretical evidence that the assignment variable cut-off is used uniquely to determine the exposure of interest. It can also be checked whether other exposures in question show discontinuity at the cut-off. For example, Bommer et al. (31) used 34 completed weeks of gestation as the cut-off for routine probiotics supplementation for neonates, and checked for the discontinuities in alternative treatments, like antibiotics, analgesics, and several other treatments. Similarly, Daysal et al. (28) in the study comparing obstetrician vs. midwife delivery supervision at the cut-off of 37 gestational weeks verified additional discontinuities in the use of vacuum/forceps during delivery, admission to NICU and hospital vs. home delivery. As shown in Table 1, in many DOHaD research contexts justifying this assumption may be challenging.

##### 3.2.2.4 Exchangeability around the assignment variable cut-off

**Figure 4:**
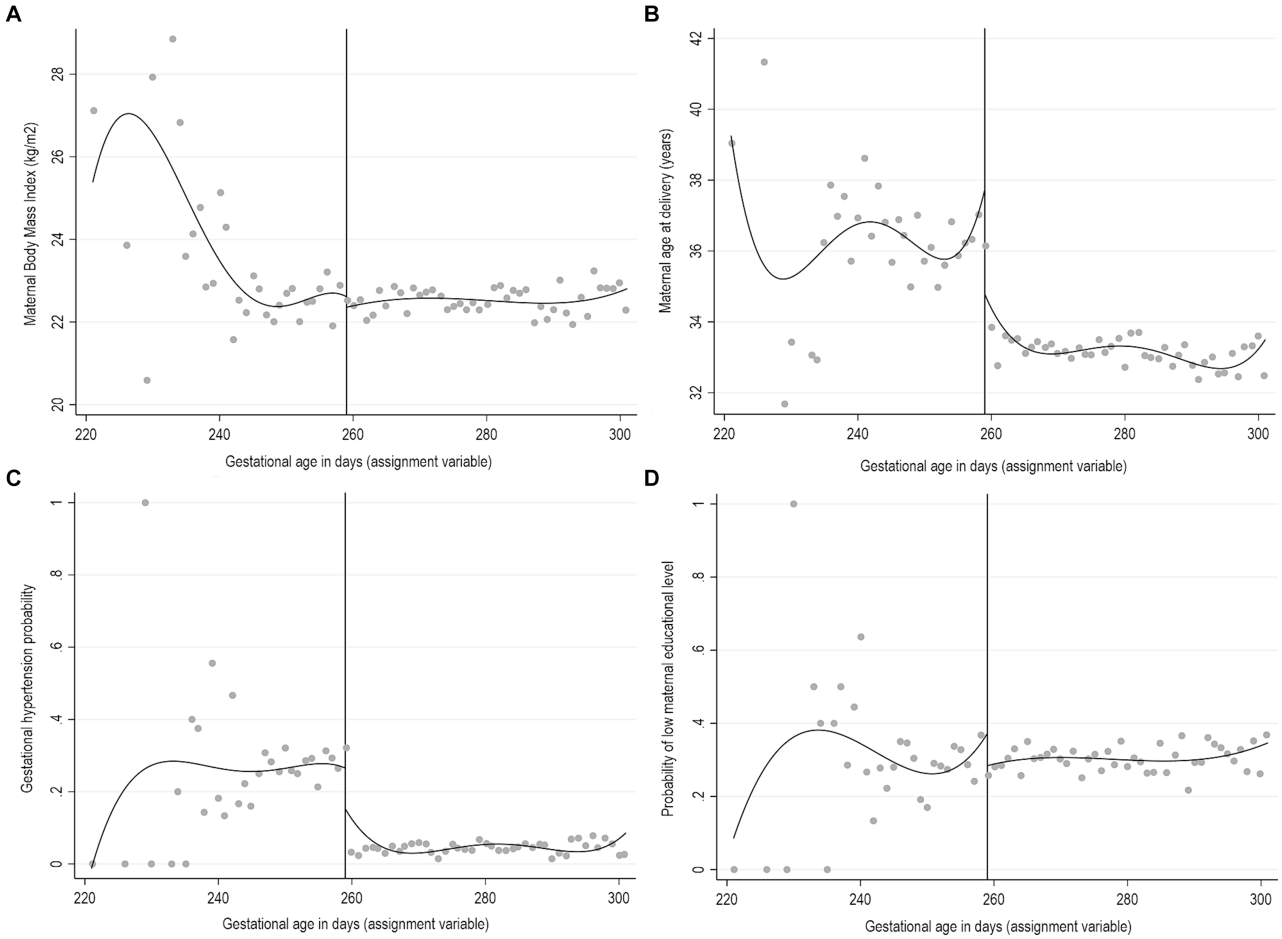
Graphical representation of RDD for predetermined covariates for a hypothetical example of gestational age as an assignment variable for obstetrician’s instead of midwife’s supervision of delivery. Simulated data. **(A)** Maternal body mass index (BMI), **(B)** Maternal age at delivery, **(C)** Gestational hypertension, **(D)** Low maternal educational level.

The exchangeability assumption, in the RDD settings also called the continuity assumption, implies that individuals just above and below the cut-off are similar with respect to the distribution of observed and unobserved factors, except for the exposure, and thus they have the same potential outcome for either exposure level (9). Although exchangeability cannot be tested, it is possible to check if the observed baseline characteristics have a similar distribution above and below the cut-off. A graphical inspection involves a series of simple plots of the relationship between the observed baseline covariates not affected by the exposure and the assignment variable. For example, in our previously described simulated example of the effect of the supervision of deliveries on the infant health outcomes, we can examine the distribution of the observed maternal baseline characteristics around the 259 days of gestation (Figure 4). In this hypothetical example, the observed discontinuity in maternal age and gestational hypertension probability at the cut-off indicate the imbalance of predetermined covariates that may threaten the validity of the design. All five previous studies that used gestational age as the assignment variable performed similar checks on the pre-exposure maternal and pregnancy characteristics (22, 28–31). It is also advisable to perform formal tests, for example using nonparametric local polynomial techniques within the continuity framework (9, 10, 12–14). Both the graphical inspections and the formal testing should be interpreted with caution when there are several observed relevant covariates to assess, as some discontinuities may be observed by random chance only.

In situations with imbalances at the cut-off in the distribution of the baseline covariates, which are likely to be important determinants of the outcome, RDD fails to provide a valid estimate of the true effect of an exposure of interest. Although covariate adjustment can be incorporated in the RDD estimations, it cannot be used to improve the validity of the design but only to enhance the efficiency of the local polynomial RD estimator (34, 35).

##### 3.2.2.5 Local monotonicity assumption

The fuzzy RDD estimates the local average treatment effect (LATE) (36), which is the average treatment effect for the compliers. Its identification requires that additional monotonicity or “no defiers” assumption (37) is met. This assumption, which is characteristic of the IV design, implies a monotonic relationship between the variable indicating the assignment and the exposure. Being untestable, the plausibility of this assumption should be investigated by knowledge of the context and observed data patterns (37). However, this was rarely done in previous RDD studies on health effects of early life exposures.

#### 3.2.3 Sensitivity analyses and diagnostic checks

The sensitivity of the results to small variations in data and estimation procedure can be verified with several additional, strongly advised, sensitivity analyses and checks that are briefly summarized below.

##### 3.2.3.1 Discontinuities in average outcomes at values other than the assignment variable cut-off

The RDD analysis consists in visually depicting and estimating a discontinuity in the outcomes of interest at the two sides of the assignment variable cut-off (Supplementary Figure S4). One of the RDD robustness checks is the comparison of the effects for true and artificial (placebo) cut-offs in the assignment variable. Any discontinuity in artificially imposed cut-offs is an indication of potentially invalid RDD. This can be verified empirically by replacing the true cut-off value by different values of the assignment variable where exposure should not change, and by repeating both the graphical and the estimation analysis, as presented in two articles by Daysal et al. (22, 28).

##### 3.2.3.2 Sensitivity to the selection of the window around the assignment variable cut-off

The most frequently used RDD estimation methods are non-parametric or local methods that consider only observations in a selected window around the cut-off. Optimal bandwidth size can be selected either *a priori* or by data-driven algorithms (38). In practice, the bandwidth size depends on data availability around the cut-off. Ideally, one would like to use a very narrow window around the cut-off, but this comes at the cost of less precise estimates (39). Sensitivity analysis with alternative specifications of bandwidth size to check the robustness of the estimated effects is a standard in RDD (22, 28–31).

##### 3.2.3.3 Sensitivity to observations near the cut-off

Even if there is no evidence of manipulation in the assignment variable, the observations very near the cut-off are likely to be the most influential when fitting local polynomials. The sensitivity “donut hole” method consists of repeating the analysis on different subsamples where observations are removed in a symmetric distance around the cutoff, starting with the closest and then increasing the distance around the cut-off in the attempt to understand the sensitivity of the results to those observations (40, 41). Sensitivity donut hole analysis was presented in two studies focused on comparing obstetrician vs. midwife delivery supervision at the cut-off of 37 gestational weeks (22, 28).

## 4 Advantages and limitations of RDD in the context of DOHaD research

RDD has several advantages over other non-experimental study designs. Its estimates and validity checks can be easily presented using simple graphical representations that improve transparency and integrity of the results. The interpretation of the results is intuitive and straightforward. RDD can provide valid causal estimates under weaker assumptions compared to other non-experimental study designs, and many of the assumptions can be assessed empirically. When an assignment variable for an exposure is found, it is possible to identify a (local) causal effect of that exposure for multiple outcomes and, if the assignment variable is not context-specific, in multiple populations.

Most of the previous RDD studies on the health effects of exposures acting early in life were conducted on data from registries and administrative databases, which often lack important details and individual-level data. With a recent exception (42), the existing birth cohorts, which collect a plenty of detailed data on pregnancy outcomes, newborn, infant, and later childhood health outcomes and represent a unique and valuable source of data for DOHaD research, have not been exploited using RDD. There are several reasons for this. The external validity of RDD studies is often considered the main caveat, as the causal effect estimate is limited to the subpopulation of individuals at the assignment variable cut-off. In the sharp RDD the treatment effect is interpreted as the average treatment effect at the cut-off, and only in some RDD settings and with an additional conditional independence assumption (by conditioning on other predictors of outcome besides the assignment variable) it can be generalized and approximated to the average treatment effect (43). The LATE obtained in a fuzzy RDD is even less generalizable because it is inferred only to the subpopulation of compliers at the cutoff. In addition, RDD often addresses very setting-specific research questions (e.g., the effect of country-specific policies) that cannot be always replicated in different populations. The utility of RDD also depends on the practical and clinical relevance of the cut-off being studied.

The estimation in RDD implies that we need adequate power for estimating the regression line on both sides of the cut-off, i.e., a lot of observations near the cut-off. While in the context of registry-based research this may be feasible, the existing birth cohorts, although rich with individual-level data, often lack a sufficient sample size. The exact power will depend on the distribution of the assignment variable, the bandwidth chosen for the analysis, and whether the design is sharp or fuzzy (needs more power). If we assume that the assignment variable is normally distributed, the percentage of the original birth cohort available for RDD for different assignment variable cut-offs and bandwidths (expressed in terms of standard deviations [SD] from the mean, and +/− SDs, respectively) is shown in Table 3. For example, if the cut-off is at 0.5 SD from the mean of the assignment variable and the bandwidth is +/− 0.5 SD, then only 34% of the original cohort is used for the analysis.

**Table 3:** Percentage of the original study available for RDD for different bandwidths and cut-offs, assuming normally distributed assignment variable *X*.

Assignment variable cut-off	Assignment variable bandwidth around the cut-off	Percentage of the original study available for RDD
Mean (*X*)	± 0.5 SD	38.3%
Mean (*X*) ± 0.5 SD	± 0.5 SD	34.1%
Mean (*X*) ± 1 SD	± 0.5 SD	24.2%
Mean (*X*) ± 2 SD	± 0.5 SD	6.1%
Mean (*X*)	± 1 SD	68.3%
Mean (*X*) ± 0.5 SD	± 1 SD	62.5%
Mean (*X*) ± 1 SD	± 1 SD	47.7%
Mean (*X*) ± 2 SD	± 1 SD	15.7%

*X*, assignment variable; SD, standard deviation.

Finally, despite an increasing popularity of RDD in studying health effects of early life exposures, settings other than policy and program evaluations are still relatively rare. Still, the assignment variables’ cut-offs for exposures applied in previous studies can be used, if appropriate, in other settings and with additional outcomes to address different research questions.

Although providing guidelines and recommendations on how to apply RDD in the context of DOHaD research is beyond our scope, we underline some key points that need to be considered when planning a RDD study in this context. First, researchers should look for large databases, drawing on administrative data or international cohort collaborations, and check that there are enough events among subjects around the cut-off. This is a crucial step, also considering the current international difficulties in data access and data sharing. For example, to check the feasibility of a RDD study aiming at, say, estimating the effect of an exposure on a specific outcome using gestational age as the assignment variable within the context of an international birth cohort collaboration, a researcher would need first to ask all participating cohorts to report the number of events in the children born in the week before and the week after the cut-off. A second key point regards the assignment variables. As reviewed in this article, a number of assignment variables, cut-offs and related exposures and/or interventions have been identified and used in previous RDD studies focused on health effects of early life exposures. These discontinuities have often been employed in multiple studies and with different databases, validating repeatedly the robustness of the RDD settings and verifying the underlying assumptions. We suggest drawing on previous experience to exploit already identified discontinuities, if not too setting specific, for the study of multiple outcomes. The identification of new discontinuities is more complex and may even be considered as a separate specific research objective. Finally, as one of the main strengths of the RDD approach is the possibility to assess empirically several of the underlying assumptions, it is important to verify that the data required to conduct the corresponding sensitivity analyses are available in the study database and/or are included in the plans to collect or to harmonize new data.

## 5 Conclusion

The regression discontinuity design is a powerful approach for causal inference in DOHaD research. Its widespread use in studying health effects of early life exposures has been hampered by the limited external validity of RDD studies and the rarity of settings outside of program and policy evaluation. The identification of discontinuities and RDD principles should be introduced to researchers who should exploit the utilities of this design in the existing population registries and birth cohorts whenever the setting and research question allow.

## Author contributions

MP: Conceptualization, Formal analysis, Methodology, Writing – original draft, Writing – review & editing. DZ: Methodology, Supervision, Writing – review & editing. KT: Methodology, Writing – review & editing. LR: Conceptualization, Funding acquisition, Methodology, Supervision, Writing – review & editing.
