# Supplementary material for: Regression discontinuity design for the study of health effects of exposures acting early in life
Source: Front Public Health. 2024 Apr 19;12:1377456. doi: 10.3389/fpubh.2024.1377456 (PMC11066219; doi:10.3389/fpubh.2024.1377456)
Supplement: Supplementary file 1 [file Data_Sheet_1.pdf]

Regression discontinuity design for the study of health effects of exposures acting early in life

Maja Popovic, Daniela Zugna, Kate Tilling, Lorenzo Richiardi

Supplementary Material

Supplementary Methods. Simulation study……………………………………………………………………… 2

Supplementary Methods. Identification of studies focused on health effects of exposures acting early in life…….. 3

Supplementary Table S1. PubMed search strategy……………………………………………………………….…4

Supplementary Table S2. Regression discontinuity studies focused on health effects of early life exposures identified from a previous systematic review on the use of RDD in health research (*Hilton Boon Epidemiology 2021, updated until March 2019*) ……………………………………………………………………………………………… 5

Supplementary Table S3. Regression discontinuity studies focused on health effects of early life exposures identified from a new PubMed search (January 1, 2019 - January 1, 2024) ……………………………………………… 9

Supplementary Figure 1. Summary of the settings in which the identified RDD studies on health effects of early life exposures were conducted, by the presence of the articles in PubMed …………………………………………… 12

Supplementary Figure 2. Summary of the assignment variables previously used in RDD studies on health effects of early life exposures, by the presence of the articles in PubMed ……………………………………………………. 13

Supplementary Figure 3. Graphical representation of the assignment variable manipulation ……………………. 14

Supplementary Figure 4. Discontinuity in the outcome at the assignment variable cut-off ……………………… 15

References ……………………………………………………………………………………………………... 16

Supplementary Methods

Simulation study

We simulated data motivated by the study of Daysal et al.[1], which investigated the effect of the obstetrician supervision of deliveries on the short-term infant health outcomes, using a rule of 37 gestational weeks (259 days) at delivery for obstetrician instead of midwife delivery supervision. Specifically, we created a dataset with 5000 observations and the following variables:

- *Gestational age in days* (*gage*) with a mean of 274 days and standard deviation of 13 days. We generated binomial discrete random variable to approximate continuous normal variable (from https://fanwangecon.github.io/R4Econ/statistics/discrandvar/htmlpdfr/fs_disc_approx_cts.html) translating between the normal random variable's parameters (mean and standard deviation) and the binomial discrete random variable's parameters (number of trials and the success probability). We restricted the dataset to generate the plots only to randomly generated observations with gestational age of less than 301 days (43 weeks).
- *Fully compliant assignment rule for a sharp RDD* as a binary variable *D* indicating whether the observations are below/equal or above 259 days of gestational age.
- *Non fully compliant assignment rule for a fuzzy RDD* as a binary variable indicating probability that depends on *x* (normally distributed random variable with mean zero and standard deviation one) through a logistic regression equation $y = 0.8 + x$ for gestational age ≤259 days, and $y = -2 + x$ for gestational age >259 days.
- *Manipulated gestational age at birth* – a variable manipulated at the cut-off of 259 days as *1.5*D + gage*.
- *Maternal body mass index* as a normally distributed random variable with a mean of 22.5 kg/m^2 and a standard deviation of 3.9 kg/m^2.
- *Maternal age at delivery* as a normally distributed random variable with a mean of 33.2 years and a standard deviation of 4.4 years. This variable was further modified by adding 3 years to observation with gestational age ≤ 259 days of gestation in order to simulate discontinuity of a predetermined variable.
- *Gestational hypertension* as a random binary variable with a probability of 4% and then modified through equation *invlogit(ln(0.4) + ln(0.75)*x)* for gestational age ≤259 days, where x is a normally distributed random variable with mean zero and standard deviation one.
- *Low maternal educational level* as a random binary variable with a probability of 30%.
- *Emergency caesarean section* as a random binary variable with a probability of 10% and then modified through equation *invlogit(ln(0.35) + ln(0.60)*x)* for gestational age >259 days, where x is a normally distributed random variable with mean zero and standard deviation one.
- Child weight at 6 months of age as a normally distributed random variable linearly dependent of *gage* with a mean of 7.5kg and a standard deviation of 1kg.

All the simulations and graphs were generated using statistical software STATA (release 15, StataCorp 2017, College Station, TX: StataCorp LLC).

Identification of studies focused on health effects of exposures acting early in life

From the studies provided in the systematic review by Hilton Boon et al.[2] we identified studies on health effects of exposures acting in fetal life, infancy, childhood, or adolescence with the aim of understanding the potential of promoting the use of RDD in DOHaD research using register-based studies and the existing birth cohort consortia. These studies were published in the period 1960-2019. We updated the search until January 1, 2024, using PubMed, with a more specific search strategy detailed below in Supplementary Table S1.

Of 325 studies from the previous systematic review by Hilton Boon et al.[2] we considered 74 studies focused on health effects of early life exposures (Supplementary Table S2, Figure 1). The additional PubMed search identified 132 studies, of which 51 were considered relevant for the current review (Supplementary Table S3, Figure 1). We did not consider studies that analysed exposures acting in early life but did not focus on their effects on health outcomes. Overall, we identified 125 RDD studies on health effects of early life exposures (Figure 1).

Supplementary Table S1. PubMed search strategy

Database	Date	Search strategy	Filter	Number of results
PubMed	01/01/2024	**("regression discontinuity" OR "regression discontinuity design") AND (child* OR offspring OR newborn OR infan* OR neonatal OR perinatal OR adolescen* OR teenag* OR mater* OR mother OR pater* OR father OR parent* OR pregnan* OR birth)**<br>(("regression discontinuity"[All Fields] OR "regression discontinuity design"[All Fields]) AND ("child*"[All Fields] OR ("offspring"[All Fields] OR "offspring s"[All Fields] OR "offsprings"[All Fields]) OR ("infant, newborn"[MeSH Terms] OR ("infant"[All Fields] AND "newborn"[All Fields]) OR "newborn infant"[All Fields] OR "newborn"[All Fields] OR "newborns"[All Fields] OR "newborn s"[All Fields]) OR "infan*"[All Fields] OR ("infant, newborn"[MeSH Terms] OR ("infant"[All Fields] AND "newborn"[All Fields]) OR "newborn infant"[All Fields] OR "neonatal"[All Fields] OR "neonate"[All Fields] OR "neonates"[All Fields] OR "neonatality"[All Fields] OR "neonatals"[All Fields] OR "neonate s"[All Fields]) OR ("perinatal"[All Fields] OR "perinatally"[All Fields] OR "perinatals"[All Fields]) OR "adolescen*"[All Fields] OR "teenag*"[All Fields] OR "mater*"[All Fields] OR ("mother s"[All Fields] OR "mothered"[All Fields] OR "mothers"[MeSH Terms] OR "mothers"[All Fields] OR "mother"[All Fields] OR "mothering"[All Fields]) OR "pater*"[All Fields] OR ("father s"[All Fields] OR "fathered"[All Fields] OR "fathers"[MeSH Terms] OR "fathers"[All Fields] OR "father"[All Fields] OR "fathering"[All Fields]) OR "parent*"[All Fields] OR "pregnan*"[All Fields] OR ("birth s"[All Fields] OR "birthed"[All Fields] OR "birthing"[All Fields] OR "parturition"[MeSH Terms] OR "parturition"[All Fields] OR "birth"[All Fields] OR "births"[All Fields]))) AND (2019:2023[pdat])<br>***Translations***<br>offspring: "offspring"[All Fields] OR "offspring's"[All Fields] OR "offsprings"[All Fields]<br>newborn: "infant, newborn"[MeSH Terms] OR ("infant"[All Fields] AND "newborn"[All Fields]) OR "newborn infant"[All Fields] OR "newborn"[All Fields] OR "newborns"[All Fields] OR "newborn's"[All Fields]<br>neonatal: "infant, newborn"[MeSH Terms] OR ("infant"[All Fields] AND "newborn"[All Fields]) OR "newborn infant"[All Fields] OR "neonatal"[All Fields] OR "neonate"[All Fields] OR "neonates"[All Fields] OR "neonatality"[All Fields] OR "neonatals"[All Fields] OR "neonate's"[All Fields]<br>perinatal: "perinatal"[All Fields] OR "perinatally"[All Fields] OR "perinatals"[All Fields]<br>mother: "mother's"[All Fields] OR "mothered"[All Fields] OR "mothers"[MeSH Terms] OR "mothers"[All Fields] OR "mother"[All Fields] OR "mothering"[All Fields]<br>father: "father's"[All Fields] OR "fathered"[All Fields] OR "fathers"[MeSH Terms] OR "fathers"[All Fields] OR "father"[All Fields] OR "fathering"[All Fields]<br>birth: "birth's"[All Fields] OR "birthed"[All Fields] OR "birthing"[All Fields] OR "parturition"[MeSH Terms] OR "parturition"[All Fields] OR "birth"[All Fields] OR "births"[All Fields]	From January 1, 2019	132

Supplementary Table S2. Regression discontinuity studies focused on health effects of early life exposures identified from a previous systematic review on the use of RDD in health research (*Hilton Boon, Epidemiology 2021,*[2] *updated until 2019*)

AUTHOR	SETTING	EXPOSURE/INTERVENTION	ASSIGNMENT VARIABLE	OUTCOME	PRESENT IN PUBMED
Almond (2010)[3]	Clinical	Neonatal intensive care	Birthweight	Infant mortality	Yes
Del Bono (2011)[4]	Clinical	Committee on Safety of Medicines health warning on combined oral contraceptives and risk of venous thromboembolism	Calendar time	Neonatal health outcomes	No
Bharadwaj (2013)[5]	Clinical	Neonatal intensive care, extra medical attention and lung surfactant therapy	Birthweight	Child academic achievement, mortality	Yes
Daysal (2013)[1]	Clinical	Obstetrician supervision of preterm birth	Gestational age	Seven- and 28-day mortality, Apgar score	No
Jensen (2015)[6]	Clinical	Publication of guidelines on Caesarean section for breech births	Calendar time	Apgar score, physician visits, hospital admissions, complications, infections	Yes
Garrouste (2011)[7]	Healthcare/Insurance	Reimbursement eligibility for amniocentesis	Down syndrome risk score	Birthweight, preterm birth	Yes
Almond (2011)[8]	Healthcare/Insurance	Length of hospital stay	Clock time	Newborn health	No
De La Mata (2012)[9]	Healthcare/Insurance	Medicaid	Family income	Healthcare utilization, health status, obesity, school sickness absence	Yes
Koch (2013)[10]	Healthcare/Insurance	Public health insurance for children	Family income	Healthcare utilization and expenditure	Yes
Camacho (2013)[11]	Healthcare/Insurance	Subsidized Regime health insurance for the poor	Poverty index	Birthweight, Apgar score	No
Palmer (2015)[12]	Healthcare/Insurance	Public health insurance for preschool children	Age	Healthcare utilization, expenditure, substitution (crowd out)	Yes
Han (2016)[13]	Healthcare/Insurance	Children's Medical Subsidy Program	Age	Healthcare utilization and expenditure	No
Bhowmick (2016)[14]	Healthcare/Insurance	Community health worker programme	Population	Child health outcomes	No
Laughery (2016)[15]	Healthcare/Insurance	Health Professional Shortage Area designation	General practitioners per 10,000 population	Neonatal health	No
Lee (2017)[16]	Healthcare/Insurance	Medicaid plan (fee for service vs managed care)	Birthweight	Hospital readmission, length of stay, mortality	No
Bernal (2017)[17]	Healthcare/Insurance	Social health insurance	Household welfare index	Children’s checkups/ healthcare utilization	No
Rashad (1992)[18]	Preventive/Vaccination programs	National Control of Diarrheal Diseases Project	Calendar time	Infant mortality	No
Schanzenbach (2009)[19]	Preventive/Vaccination programs	National School Lunch Program	Income to poverty ratio	Child obesity	No

AUTHOR	SETTING	EXPOSURE/INTERVENTION	ASSIGNMENT VARIABLE	OUTCOME	PRESENT IN PUBMED
Ziegelhöfer (2012)[20]	Preventive/Vaccination programs	Rural water supply and hygiene education programme	Investment cost per inhabitant	Diarrheal disease in children under 5 years	No
Peckham (2012)[21]	Preventive/Vaccination programs	National School Lunch Program	Family income to poverty ratio	Obesity (BMI, waist-to hip ratio, percentage body fat)	No
Meller (2014)[22]	Preventive/Vaccination programs	PANN2000 food supplementation and health check programme	Poverty index	Child mortality	No
Yan (2014)[23]	Preventive/Vaccination programs	Minimum cigarette purchase age	Maternal age at conception	Infant health	No
Dykstra (2015)[24]	Preventive/Vaccination programs	Gavi vaccination aid programme	Multiple per capita national income	Child mortality	No
McMahon (2015)[25]	Preventive/Vaccination programs	School-based food intervention to reduce radionuclide exposure	Calendar time	Blood markers, anaemia, respiratory and immune diseases	Yes
Almond (2016)[26]	Preventive/Vaccination programs	Obesity report cards for schoolchildren	Body mass index (BMI)	BMI and weight	Yes
Gertner (2016)[27]	Preventive/Vaccination programs	Home visits to promote nutritional knowledge and child health	Distance to geographical boundary	Anthropometric measures	No
Bakolis (2016)[28]	Preventive/Vaccination programs	Smoking ban	Calendar time	Birth outcomes	Yes
Billings (2018)[29]	Preventive/Vaccination programs	Interventions for elevated blood lead levels in children	Blood lead levels	Adolescent Antisocial Behaviour Index	No
Ludwig (2007)[30]	Social and welfare programs	Head Start	County-level poverty index	Child mortality	No
Rosero (2011)[31]	Social and welfare programs	Early childhood programmes (home visits and childcare centres for poor families)	Proposal quality score	Child health measures	No
Medina (2013)[32]	Social and welfare programs	Unemployment Subsidy and retraining	Welfare index	Children's weight, height, BMI, Apgar score	No
You (2013)[33]	Social and welfare programs	Formal microcredit	Predicted probability of borrowing microcredit	Child malnutrition (BMI, anaemia, zinc deficiency)	No
Carneiro (2014)[34]	Social and welfare programs	Head Start	Family income	Multiple health, cognitive and behavioural measures	No
Cogneau (2015)[35]	Social and welfare programs	National boundaries	Distance from border	Children's height-for age	No
El-Kogali (2015)[36]	Social and welfare programs	Community development programme	District poverty level	Child growth and nutrition	No
Beuchert (2016)[37]	Social and welfare programs	Maternity leave policy change	Calendar time	Child hospital visits	No

AUTHOR	SETTING	EXPOSURE/INTERVENTION	ASSIGNMENT VARIABLE	OUTCOME	PRESENT IN PUBMED
Deepti Thomas (2016)[38]	Social and welfare programs	National Rural Employment Guarantee Act	State development index	Child mortality, school enrolment and dropping out, reading and math skills.	No
You (2016)[39]	Social and welfare programs	Access to microcredit	Propensity to borrow from rural microcredit schemes	Parental report of child health	Yes
Moreno (2017)[40]	Social and welfare programs	Human Development Bonus (conditional cash transfer)	Household poverty index	Child chronic stunting	No
Cattaneo (2017)[41]	Social and welfare programs	Head Start	County-level poverty index	Child mortality	Yes
Tang (2017)[42]	Social and welfare programs	Head Start	Calendar time	Child cognitive development	No
Guldi (2018)[43]	Social and welfare programs	Supplemental Security Income benefit	Birthweight	Infant mortality, child motor skill development	No
Rahman (2018)[44]	Social and welfare programs	Safe motherhood scheme (conditional cash transfer)	Parity	Use of child health services	Yes
Deutscher (2018)[45]	Social and welfare programs	Birth shifting in response to baby bonus policy	Calendar time	Birthweight, gestation length, child developmental score	No
Garcia Hombrados (2018)[46]	Social and welfare programs	Increase in legal age of marriage for women	Age	Infant mortality	No
Gormley (2005)[47]	Education	Universal prekindergarten program	Time (date of birth)	School readiness	Yes
Wong (2008)[48]	Education	State prekindergarten programme	Time (date of birth)	Children's cognitive skills/school readiness	No
Coburn (2009)[49]	Education	Prekindergarten programme	Age	School readiness skills	No
Lindeboom (2009)[50]	Education	Additional year of schooling	Time (year of birth)	Child height, weight, morbidity	Yes
Zhang (2009)[51]	Education	Years of formal schooling	Age	Children's bodyweight	No
Elder (2010)[52]	Education	School starting age	Time (date of birth)	ADHD symptoms, diagnosis and treatment	Yes
Evans (2010)[53]	Education	School starting age	Time (date of birth)	ADHD diagnosis and treatment	Yes
Lipsey (2011)[54]	Education	Voluntary pre-kindergarten programme	Time (date of birth)	School readiness	No
McCrary (2011)[55]	Education	School starting age	Time (date of birth)	Birthweight, prematurity	Yes
Anderson (2011)[56]	Education	Years of early primary education	Time (date of birth)	Children's BMI	Yes
Nakamura (2012)[57]	Education	Maternal schooling	Time (month and year of birth)	Children's weight	No
Weiland (2013)[58]	Education	Public Schools prekindergarten programme	Date of birth	Cognitive, executive function and emotional development	Yes
Jakobsson (2013)[59]	Education	Class size	Class size	Adolescent mental health and wellbeing	No
Ankara (2015)[60]	Education	Extension of compulsory schooling	Time (date of birth)	Child mortality	No

AUTHOR	SETTING	EXPOSURE/INTERVENTION	ASSIGNMENT VARIABLE	OUTCOME	PRESENT IN PUBMED
Chen (2015)[61]	Education	School starting age	Age	Inattentive/hyperactive behaviour	No
Grépin (2015)[62]	Education	1980 School Reform in Zimbabwe	Age	Child mortality	Yes
Makate (2016)[63]	Education	Universal Primary Education policy	Age	Neonatal and child mortality	Yes
Schwandt (2016)[64]	Education	School starting age	Age	ADHD prevalence and medications	No
Ali (2018)[65]	Education	Parental education	Time (date of birth)	Child mortality	Yes
Dee (2018)[66]	Education	Delay starting school by one year	Age	Child emotional, conduct, hyperactivity, peer problems, prosocial behaviour	Yes
Makate (2018)[67]	Education	1980 School Reform in Zimbabwe	Calendar year	Child height-for-age and weight-for-age	No
Keats (2018)[68]	Education	Universal primary education programme	Time (year of birth)	Height- and weight-for age, stunting, anaemia, infant mortality	No
Chay (2003)[69]	Shock events, environmental and social factors	Clean Air Act Amendments (1970)	Total suspended particulates	Infant mortality	No
Yang (2008)[70]	Shock events, environmental and social factors	Clean Air Act Amendments (1970)	Total suspended particulates	Infant mortality	No
Dell (2010)[71]	Shock events, environmental and social factors	The mita (forced labour system)	Latitude and longitude	Stunted growth in children	No
Huang (2013)[72]	Shock events, environmental and social factors	Great Famine 1959-61 (primary education)	Time (year of birth)	Cognitive functioning in adults	Yes
Sotomayor (2013)[73]	Shock events, environmental and social factors	In-utero exposure to natural disasters	Time (year of birth)	Hypertension, diabetes, high cholesterol in adulthood	Yes
Bhalotra (2014)[74]	Shock events, environmental and social factors	Rise in share of elected officials who are Muslim	Vote margin in close elections	Neonatal and infant mortality	No
Zhong (2014)[75]	Shock events, environmental and social factors	Number of siblings	Time (year of birth)	Child height, self-assessed health, BMI	No

Supplementary Table S3. Regression discontinuity studies focused on health effects of early life exposures identified from a PubMed search (January 1, 2019 - January 1, 2024)

AUTHOR	SETTING	EXPOSURE/INTERVENTION	ASSIGNMENT VARIABLE	OUTCOME
Daysal (2019)[76]	Clinical	Obstetrician supervision of preterm birth	Gestational age	Neonatal mortality
Hutcheon (2020)[77]	Clinical	Antenatal corticosteroid administration	Gestational age	Child development score
Brilli (2020)[78]	Clinical	Neonatal care for high birthweight newborns	Birthweight	Neonatal intensive care, use of antibiotics, infant mortality
Bommer (2020)[79]	Clinical	Routine probiotics supplementation	Gestational age	Anthropometric development, late-onset sepsis
Song (2020)[80]	Clinical	Tight glycaemic control in pregnant women diagnosed with GDM	Composite score based on oral glucose tolerance tests	Birthweight, size for gestational age, neonatal hypoglycaemia, admission to special care nursery, shoulder dystocia
Harrison (2020)[81]	Clinical	Postoperative massage in pediatric patients with complex congenital heart disease	Time	Heart rate, respiratory rate, oxygen saturation
Worsham (2021)[82]	Clinical	Opioid prescription in adolescents	Age	Opioid-related adverse events
Chyn (2021)[83]	Clinical	Early-life interventions for low-birth-weight newborns	Birthweight	Grade repetition, special education services, test scores, high school disciplinary offenses, college preparation, child Medicaid enrolment and expenditures by age 2
Holzhausen (2021)[84]	Clinical	Difference between objective and subjective evaluation of sleep duration among children	Age	Sleep duration
Furzer (2022)[85]	Clinical	Discrepancy between teacher and parent ADHD assessments	Time (date of birth)	Over- or under-diagnosis of ADHD
Kim (2022)[86]	Clinical	Overweight/obese classification	Children's BMI at earlier age	Children BMI at later age
Hutcheon (2022)[87]	Clinical	Antenatal corticosteroid administration	Gestational age	Child ADHD medication data
Tennant (2022)[88]	Clinical	Clinical diagnosis of gestational diabetes	Fasting plasma glucose	Birthweight, large for gestational age, caesarean section, shoulder dystocia, perinatal death
van der Linde (2022)[89]	Clinical	Treatment in a specialized paediatric haemato-oncology vs. adult haemato-oncology setting	Age	5-Year Survival in Acute Lymphoblastic Leukaemia
Nishioka (2021)[90]	Healthcare/Insurance	Medical expenditure after marginal cut of cash benefit	Child age	Healthcare costs, outpatient visits
Liu (2021)[91]	Healthcare/Insurance	Medicaid managed care versus Medicaid fee-for-service	Birthweight	Emergency department use and hospitalization during the first 6-12 months of life among low birthweight infants
Geiger (2021)[92]	Healthcare/Insurance	Use of prenatal healthcare services	Maternal age	Preterm birth or low birth weight, perinatal mortality

AUTHOR	SETTING	EXPOSURE/INTERVENTION	ASSIGNMENT VARIABLE	OUTCOME
Epure (2023)[93]	Healthcare/Insurance	Swiss health policy expansion fully covering illness-related costs during pregnancy	Time (date of birth)	Birthweight, gestational age, preterm, neonatal death
Fukuma (2023)[94]	Healthcare/Insurance	Cost sharing of healthcare spending for children	Age	Children outpatient and inpatient healthcare care usage
Raifman (2020)[95]	Preventive/Vaccination programs	Minimum purchaser age for the sale of handguns	Age	Adolescent suicide rate
de Chaisemartin (2021)[96]	Preventive/Vaccination programs	Newborns' BCG vaccination	Time (birth year)	COVID-19 cases and hospitalizations
Ahammer (2022)[97]	Preventive/Vaccination programs	Minimum legal drinking ages	Age	Teenage hospitalizations due to alcohol intoxication
Chuard (2020)[98]	Social and welfare programs	Policy reform on the duration of paid parental leave	Calendar time	Birthweight, gestational length, Apgar score
Alfaro-Hudak (2022)[99]	Social and welfare programs	The Supplemental Nutrition Assistance Program	Poverty-income ratio	Cardiometabolic risk factors in children and adolescents (waist circumference, HDL cholesterol, systolic blood pressure, triglycerides, glucose)
González (2022)[100]	Social and welfare programs	Universal child benefit (cash transfer)	Time (date of birth)	Birth weight, stillbirth, neonatal death in the first 24 hours, weeks of gestation
Belenko (2022)[101]	Social and welfare programs	Juvenile Drug Treatment Courts vs. Traditional Juvenile Courts	Recidivism risk and substance use level	Substances use and access to mental health services
Proshin (2023)[102]	Social and welfare programs	Maternity Capital program-a child subsidy	Time (date of birth)	Child health and developmental outcomes, chronic diseases
Reader (2023)[103]	Social and welfare programs	Health in Pregnancy Grant (universal conditional cash transfer)	Time (date of birth)	Birthweight, gestational age, prematurity
Bitler (2023)[104]	Social and welfare programs	The Special Supplemental Nutrition Program for Women, Infants, and Children	Age	Laboratory biomarkers (haemoglobin, haematocrit, and anaemia)
Rukiko (2023)[105]	Social and welfare programs	Conditional cash transfer	Proxy Means Test scores (poverty score)	Stunting (height-for-age) in children < 5 years old
Johnson (2023)[106]	Social and welfare programs	Deductibles for public drug coverage	Household income	Asthma-related medication use among low-income children
Hong (2019)[107]	Education	Universal pre-kindergarten program	Time (date of birth)	Healthcare utilization and diagnoses (physical health conditions)
Courtin (2019)[108]	Education	Education reform that raised the minimum school leaving age	Age	Biomarkers of cardiovascular, metabolic, organ and immune function
Plotnikov (2020)[109]	Education	Raising of the school leaving age	Time (year of birth)	Myopia
Butler (2020)[110]	Education	Attendance of selective schools	Test scores taken at age 11	Overall health, mental health, limitation due to health problems, number of chronic diseases, risk of death by age 60
He (2021)[111]	Education	School entry cut-off date	Time (date of birth)	Myopia

AUTHOR	SETTING	EXPOSURE/INTERVENTION	ASSIGNMENT VARIABLE	OUTCOME
Zhang (2022)[112]	Education	Compulsory Education Law	Time (month of birth)	Mean spherical equivalent refractive error and uncorrected visual acuity
Muchomba (2022)[113]	Education	Change in reform: Years of schooling	Time (year of birth)	Anaemia and BMI of reproductive age women
Ye (2022)[114]	Education	Compulsory education reforms: mandatory and free primary and lower secondary education	Time (year of birth) and province of birth	Allostatic load (measured through multiple biomarkers) in adults
Judd (2022)[115]	Education	Years of schooling	Age	Cognition (crystallized intelligence, fluid intelligence, working memory)
Broughton (2023)[116]	Education	School starting age	Time (week of birth)	Symptoms and diagnoses of mental health disorders
Ding (2023)[117]	Education	School starting age	Time (month of birth)	Myopia
Gong (2020)[118]	Shock events, environmental and social factors	Rustication program in China	Time (year of birth)	Adult physical and mental health
Fang (2020)[119]	Shock events, environmental and social factors	Foetal exposure to famine	Time (date of birth)	Anthropometric measures in adulthood
Aso (2020)[120]	Shock events, environmental and social factors	Fukushima Daiichi Nuclear Power Plant accident	Calendar time	Use of Computed Tomography in children with mild head injuries
Been (2020)[121]	Shock events, environmental and social factors	COVID-19 mitigation measures	Calendar time	Incidence of preterm birth
Buitrago (2021)[122]	Shock events, environmental and social factors	Cease-fire declared during the Colombian peace (exposure to conflict events during pregnancy)	Time (date of conception)	Stillbirth, perinatal mortality
Bakolis (2021)[123]	Shock events, environmental and social factors	Lifting of COVID-19 ‘lockdown’ policy	Calendar time	Mental health service use and mortality (including children and adolescents)
Takaku (2021)[124]	Shock events, environmental and social factors	COVID-19 school closure	Age	Child weight gain
Coma (2023)[125]	Shock events, environmental and social factors	Mandatory use of face covering masks for the control of SARS-CoV-2 in schools	Age	Incidence of SARS-CoV-2 in children
Arif (2023)[126]	Shock events, environmental and social factors	Presence of a grandparent in a household	Retirement age	Children weight for age and height for age

Supplementary Figure S1. Summary of the settings in which the identified RDD studies on health effects of early life exposures were conducted, by the presence of the articles in PubMed. *Panel A:* RDD studies on health effects of early life exposures identified from a previous systematic review (Hilton Boon, Epidemiology 2021,[2] January 1, 1960 – January 1, 2019). *Panel B:* RDD studies on health effects of early life exposures identified through a PubMed search (January 1, 2019 – January 1, 2014)

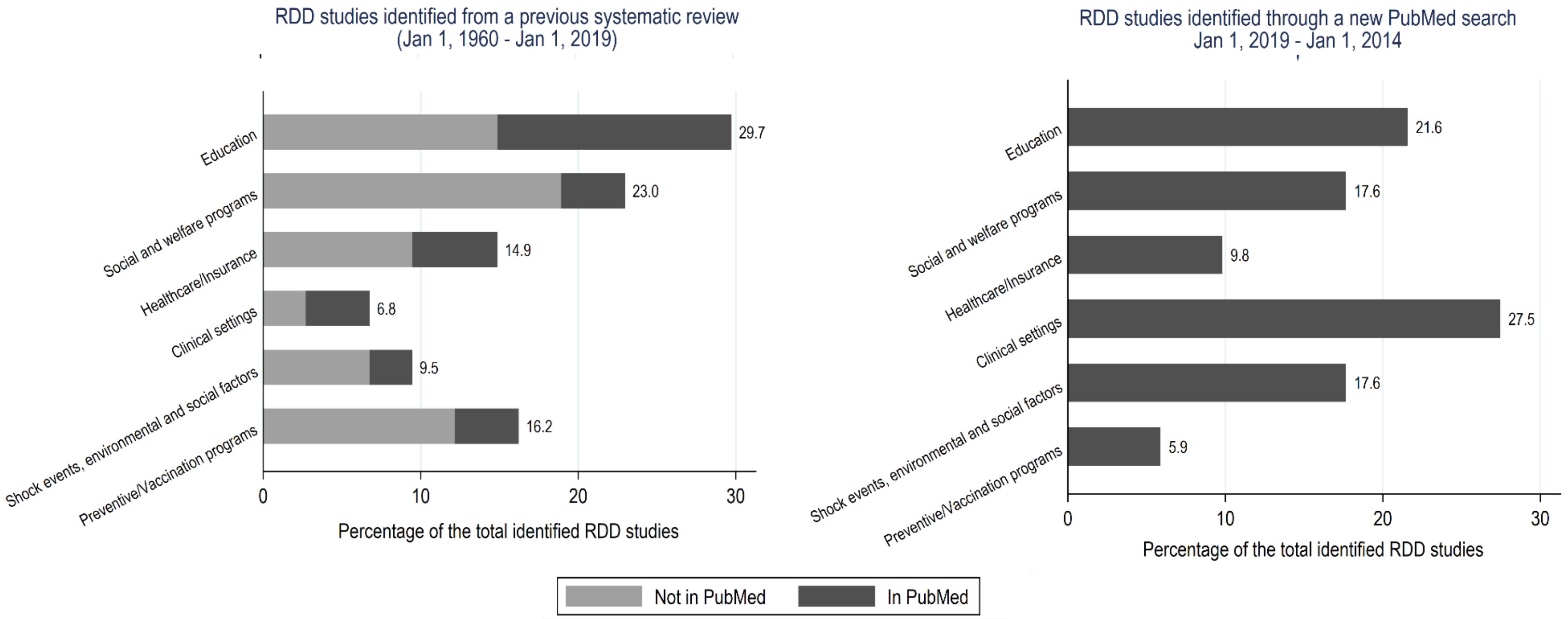

Supplementary Figure S2. Summary of the assignment variables previously used in RDD studies on health effects of early life exposures, by the presence of the articles in PubMed. *Panel A:* RDD studies on health effects of early life exposures identified from a previous systematic review (Hilton Boon, Epidemiology 2021,[2] January 1, 1960 – January 1, 2019). *Panel B:* RDD studies on health effects of early life exposures identified through a PubMed search (January 1, 2019 – January 1, 2014)

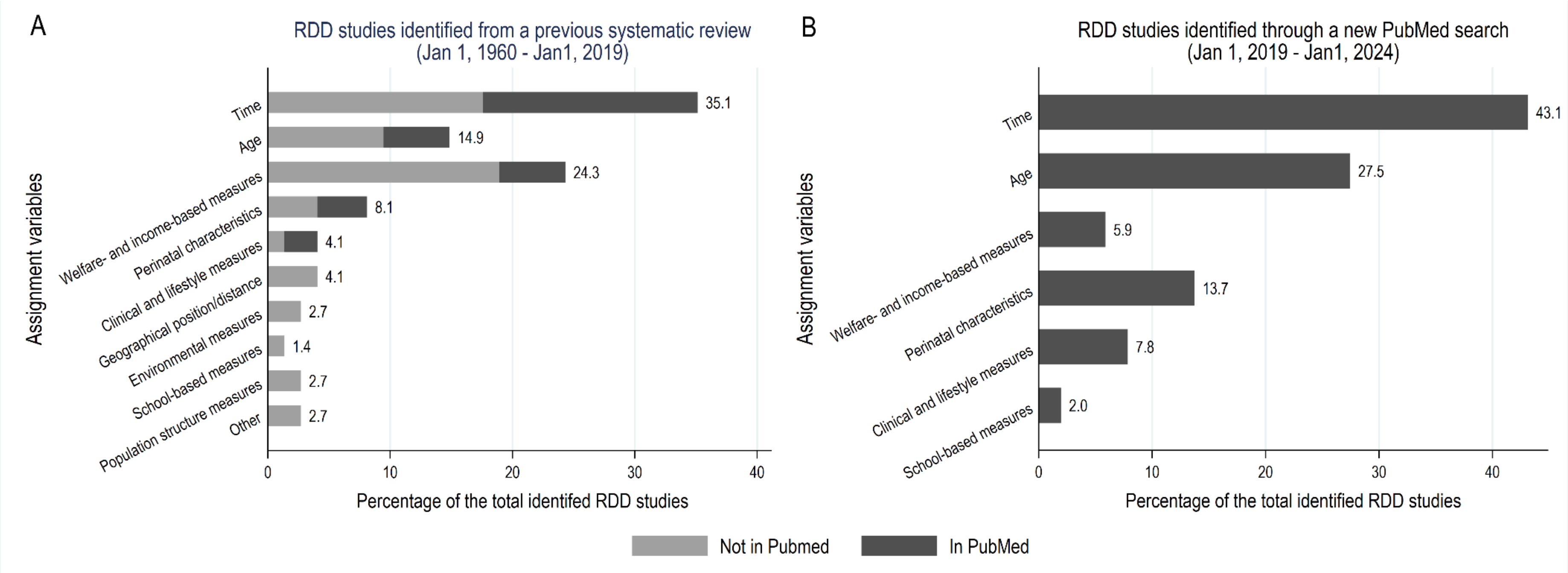

Supplementary Figure S3. Graphical representation of the assignment variable manipulation. Panel A: No evidence of manipulation in assignment variable at cut-off. Panel B: Evidence of manipulation in assignment variable at cut-off. The graphs are generated using the STATA rddensity command[127] and a simulated example of a study investigating the effect of the obstetrician's or midwife's delivery supervision, based on a rule of 37 gestational weeks (259 days) at delivery, on the short-term infant health outcomes.

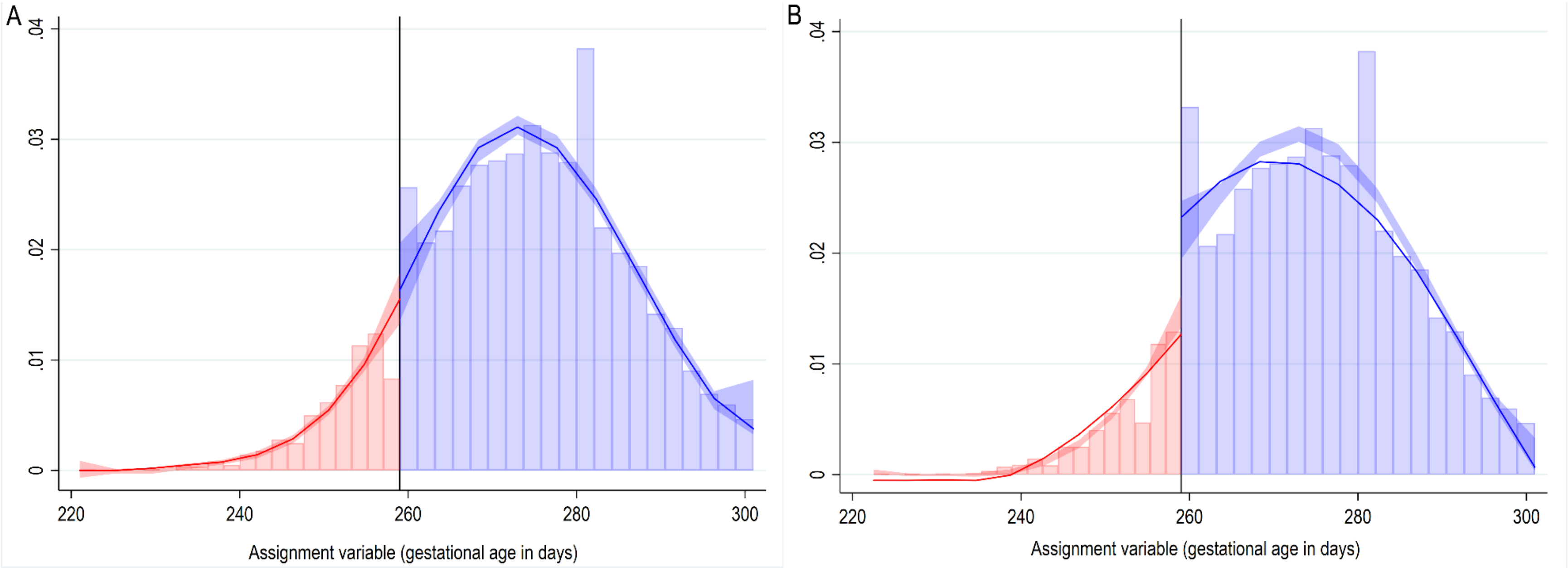

Supplementary Figure S4. Discontinuity in the outcome at the assignment variable cut-off. The graphs are generated using a simulated example of a study investigating the effect of the obstetrician's or midwife's delivery supervision, based on a rule of 37 gestational weeks (259 days) at delivery, on two perinatal and infant health outcomes: emergency caesarean section (*Panel A*) and infant weight at 6 months of age (*Panel B*). A jump in the probability of emergency caesarean section at the gestational age cut-off indicates that obstetrician's supervision of birth reduces the likelihood of emergency caesarean section (Panel A), while no effect (i.e., no discontinuity) at the cut-off was simulated for infant weight at 6 months of age (*Panel B*).

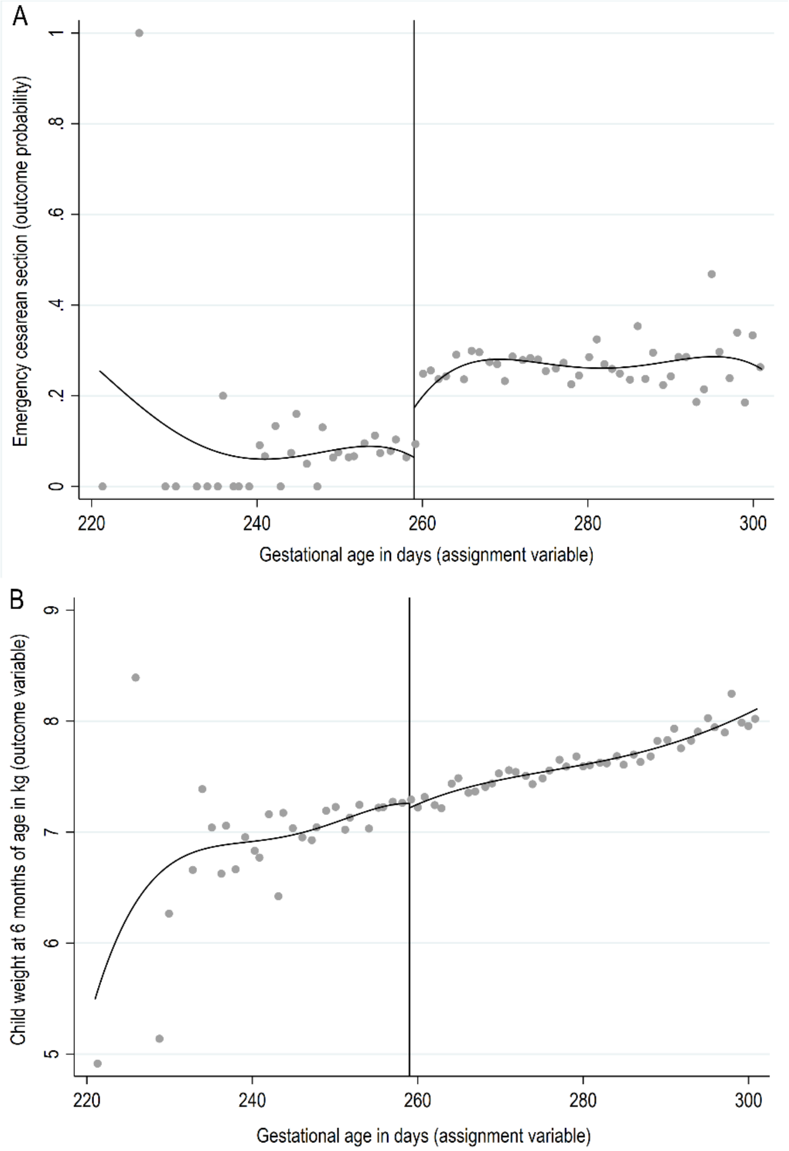

References

1. Daysal NM, Trandafir M, van Ewijk R. Returns to childbirth technologies: evidence from preterm births. *IZA Discussion Papers 7834.* Institute of Labor Economics (IZA). 2013.
2. Hilton Boon M, Craig P, Thomson H, Campbell M, Moore L. Regression Discontinuity Designs in Health: A Systematic Review [published correction appears in Epidemiology. 2021 Jul 1;32(4):e15]. Epidemiology. (2021) 32:87-93.
3. Almond D, Doyle Jr, JJ, Kowalski AE, Williams H. Estimating marginal returns to medical care: evidence from at-risk newborns. *Q J Econ.* 2010;125(2):591-634.
4. del Bono E, Francesconi M, Best NG. Health information and health outcomes: an application of the regression discontinuity design to the 1995 UK contraceptive pill scare case. ISER Working Paper Series 2011-16. Institute for Social and Economic Research. 2011.
5. Bharadwaj P, Løken KV, Neilson C. Early life health interventions and academic achievement. *Am. Econ. Rev*. 2013;103(5):1862-1891.
6. Jensen VM, Wüst M. Can Caesarean section improve child and maternal health? The case of breech babies. *J Health Econ*. 2015;39:289-302
7. Garrouste C, Le J, Maurin E. The choice of detecting Down syndrome: Does money matter? *Health Econ*. 2011;20(9):1073-1089.
8. Almond D, Doyle JJ. After midnight: A regression discontinuity design in length of postpartum hospital stays. *Am Econ J Econ Policy*. 2011;3(3):1-34.
9. de La Mata D. The effect of Medicaid eligibility on coverage, utilization, and children’s health. *Health Econ*. 2012;21(9):1061-1079.
10. Koch TG. Using RD design to understand heterogeneity in health insurance crowd-out. *J Health Econ*. 2013;32(3):599-611.
11. Camacho A, Conover E. Effects of subsidized health insurance on newborn health in a developing country. *Econ Dev Cult Change*. 2013;61(3):633-658.
12. Palmer M, Mitra S, Mont D, Groce N. The impact of health insurance for children under age 6 in Vietnam: A regression discontinuity approach. *Soc Sci Med*. 2015;145:217-226.
13. Han HW, Lien H, Yang TT. Patient cost sharing and healthcare utilization in early childhood: evidence from a regression discontinuity design. Institute of Economics, Academia Sinica, Taipei, Taiwan; 2016.
14. Bhowmick R. Three essays on economics of early life health in developing countries. PhD [dissertation]. Ann Arbour: University of Southern California; 2016.
15. Laughery S. Essays on the production of primary health care. PhD [dissertation]. University of Virginia; 2016.
16. Lee A. Essays in Applied Microeconomics. PhD [dissertation]. Columbia University; 2017.
17. Bernal N, Carpio MA, Klein TJ. The effects of access to health insurance: Evidence from a regression discontinuity design in Peru. *J Public Econ*. 2017;154:122-136.
18. Rashad H. The mortality impact of oral rehydration therapy in Egypt: re-appraisal of evidence. Baltimore, Maryland, John Hopkins University, School of Hygiene and Public Health. Institute for International Programs; 1992:135-160.
19. Schanzenbach DW. Do school lunches contribute to childhood obesity? *J Hum Resour*. 2009;44(3):684-709.
20. Ziegelhöfer Z, Panizza U, Berg G, et al. Down with diarrhea: using fuzzy regression discontinuity design to link communal water supply with health. IHEID Working Papers 05-2012, Economics Section, The Graduate Institute of International Studies, 2012.
21. Peckham JG, Kropp JD. Are national school lunch program participants more likely to be obese? Dealing with identification. Paper presented at the 2012 Annual Meeting of the Agricultural and Applied Economics Association, Seattle, Washington, August 12-14, 2012.
22. Meller M, Litschig S, Meller MW. Saving lives: Evidence from a conditional food supplementation program. *J Hum Resour*. 2014;49(4):1014-1052.
23. Yan J. The effects of a minimum cigarette purchase age of 21 on prenatal smoking and infant health. *East Econ J*. 2014;40(3):289-308.

24. Dykstra S, Glassman A, Kenny C, Sandefur J. The Impact of Gavi on vaccination rates: Regression discontinuity evidence. Washington, D.C., Center for Global Development; 2015.
25. McMahon DM, Vdovenko VY, Stepanova YI, et al. Dietary supplementation with radionuclide free food improves children's health following community exposure to 137Cesium: A prospective study. *Environ Health*. 2015;14:94.
26. Almond D, Lee A, Schwartz AE. Impacts of classifying New York City students as overweight. *Proc Natl Acad Sci U S A.* 2016;113(13):3488-3491.
27. Gertner G, Johannsen J, Martinez S, et al. Effects of nutrition promotion on child growth in El Alto, Bolivia: Results from a geographical discontinuity design. *Economia – Journal of the Latin American and Caribbean Economic Association*. 2016; 17(1);131-165.
28. Bakolis I, Kelly R, Fecht D, et al. Protective effects of smoke-free legislation on birth outcomes in England. *Epidemiology*. 2016;27(6):810-818.
29. Billings SB, Schnepel KT. Life after lead: Effects of early interventions for children exposed to lead. *Am Econ J Appl Econ*. 2018;10(3):315-344.
30. Ludwig J, Miller DL. Does Head Start improve children's life chances? Evidence from a regression discontinuity design. *Q J Econ. 2007;122(1):159-208.*
31. Rosero J, Oosterbeek H. Trade-offs between different early childhood interventions: evidence from Ecuador. Tinbergen Institute Discussion Papers 11-102/3, Tinbergen Institute; 2011.
32. Medina C, Nunez J, Tamayo JA. The unemployment subsidy program in Colombia: an assessment. IDB Working Paper Serier No. IDB-WP-369. Inter-American Development Bank, research Department; 2013.
33. You J. The role of microcredit in older children's nutrition: Quasi-experimental evidence from rural China. *Food Policy*. 2013;43:167-179.
34. Carneiro P, Ginja R. Long-term impacts of compensatory preschool on health and behavior: evidence from Head Start. *Am Econ J Econ Policy*. 2014;6(4):135-173.
35. Cogneau D, Mesplé-Somps S, Spielvogel G. Development at the border: policies and national integration in Côte D'Ivoire and its neighbors. *World Bank Econ Rev*. 2015;29(1):41-71.
36. El-Kogali S, Krafft C, Abdelkhalek T, et al. The impact of a community development and poverty reduction program on early childhood development in Morocco. Policy Research Working Paper No. 7671. World Bank, Washington, DC. 2016.
37. Beuchert LV, Humlum MK, Vejlin R. The length of maternity leave and family health. *Labour Econ*. 2016;43:55-71.
38. Deepti Thomas M. Three essays on the impact of welfare policies. PhD [dissertation]. University of Texas; 2016.
39. You J. Lending to parents and insuring children: is there a role for microcredit in complementing health insurance in rural China? *Health Econ*. 2016;25(5):543-558.
40. Moreno L. Assessing the effect of conditional cash transfers in children chronic stunting: The Human Development Bonus in Ecuador. *Analítika: Journal of Statistical Analysis / Revista de Análisis Estadístico*. 2017;13:83-131.
41. Cattaneo MD, Titiunik R, Vazquez-Bare G. Comparing inference approaches for RD designs: a reexamination of the effect of Head Start on child mortality. *J Policy Anal Manag*. 2017;36(3):643-681.
42. Tang Y, Cook TD, Kisbu-Sakarya Y, Hock H, Chiang H. The comparative regression discontinuity (CRD) design: An overview and demonstration of its performance relative to basic RD and the randomized experiment. In: Cattaneo MD, Escanciano JC, editors. Regression Discontinuity Designs: Theory and Applications. Advances in Econometrics. 2017;38:237-279.
43. Guldi M, Hawkins A, Hemmeter J, et al. Supplemental security income and child outcomes: evidence from birth weight eligibility cutoffs. Department of Economics Working Papers 2018-12, Department of Economics, Williams College; 2018.
44. Rahman MM, Pallikadavath S. How much do conditional cash transfers increase the utilization of maternal and child health care services? New evidence from Janani Suraksha Yojana in India. *Econ Hum Biol*. 2018;31:164-183.

45. Deutscher N, Breunig R. Baby bonuses: natural experiments in cash transfers, birth timing and child outcomes. *Econ Rec*. 2018;94(304):1-24.
46. Garcia Hombrados J. Empirical essays on development economics. PhD [dissertation]. University of Sussex; 2018.
47. Gormley WT, Gayer T, Phillips D, Dawson B. The Effects of universal Pre-K on cognitive development. *Dev Psychol*. 2005;41(6):872-884.
48. Wong VC, Cook TD, Barnett WS, Jung K. An effectiveness-based evaluation of five state pre-kindergarten programs. *J Policy Anal Manag*. 2008;27(1):122-154.
49. Coburn JL. The effect of Tennessee's prekindergarten programs on young children's school readiness skills: a regression discontinuity design. PhD [dissertation]. Cookeville, TN: Tennessee Technological University; 2009.
50. Lindeboom M, Llena-Nozal A, van der Klaauw B. Parental education and child health: Evidence from a schooling reform. *J Health Econ*. 2009;28(1):109-131.
51. Zhang N, Cawley J. The determinants of children's health. US: ProQuest Information & Learning; 2009.
52. Elder TE. The importance of relative standards in ADHD diagnoses: Evidence based on exact birth dates. *J Health Econ*. 2010;29(5):641-656.
53. Evans WN, Morrill MS, Parente ST. Measuring inappropriate medical diagnosis and treatment in survey data: The case of ADHD among school-age children. *J Health Econ*. 2010;29(5):657-673.
54. Lipsey MW, Farran DC, Bilbrey C, Hofer KG, Dong N, Initial results of the evaluation of the Tennessee Voluntary PreK Program [Reports – Research]. Peabody research Institute, Vanderbilt University; 2011.
55. McCrary J, Royer H. The effect of female education on fertility and infant health: evidence from school entry policies using exact date of birth. *Am Econ Rev*. 2011;101(1):158-195.
56. Anderson PM, Butcher KF, Cascio EU, Schanzenbach DW. Is being in school better? The impact of school on children's BMI when starting age is endogenous. *J Health Econ*. 2011;30(5):977-986.
57. Nakamura R. Intergenerational effect of schooling and childhood overweight. HEDG, c/o Department of Economics, University of York; 2012.
58. Weiland C, Yoshikawa H. Impacts of a prekindergarten program on children's mathematics, language, literacy, executive function, and emotional skills. *Child Dev*. 2013;84(6):2112-2130.
59. Jakobsson N, Persson M, Svensson M. Class-size effects on adolescents' mental health and well-being in Swedish schools. *Educ Econ*. 2013;21(3):248-263.
60. Ankara H. Analyses of health and health related policies in Turkey. Newcastle University; 2015.
61. Chen K, Fortin N, Phipps S. Young in class: Implications for inattentive/hyperactive behaviour of Canadian boys and girls. *Can J Econ*. 2015;48(5):1601-1634.
62. Grépin KA, Bharadwaj P. Maternal education and child mortality in Zimbabwe. *J Health Econ*. 2015;44:97-117.
63. Makate M, Makate C. The causal effect of increased primary schooling on child mortality in Malawi: Universal primary education as a natural experiment. *Soc Sci Med*. 2016;168:72-83.
64. Schwandt H, Wuppermann A. The youngest get the pill: ADHD misdiagnosis in Germany, its regional correlates and international comparison. *Labour Econ*. 2016;43:72-86.
65. Ali FRM, Elsayed MAA. The effect of parental education on child health: Quasi-experimental evidence from a reduction in the length of primary schooling in Egypt. *Health Econ*. 2018;27(4):649-662.
66. Dee TS, Sievertsen HH. The gift of time? School starting age and mental health. *Health Econ*. 2018;27(5):781-802.
67. Makate M, Makate C. Educated mothers, well-fed and healthy children? Assessing the impact of the 1980 School Reform on dietary diversity and nutrition outcomes of Zimbabwean children. *J Dev Stud*. 2018;54(7):1196-1216.
68. Keats A. Women's schooling, fertility, and child health outcomes: Evidence from Uganda's free primary education program. *J Dev Econ*. 2018;135:142-159.
69. Chay KY, Greenstone M. Air quality, infant mortality, and the Clean Air Act of 1970. Working Paper 10053: National Bureau of Economic Research; 2003.

70. Yang M. Regression discontinuity design and program evaluation. PhD [dissertation]. Berkeley: University of California; 2008.

71. Dell M. The persistent effects of Peru's Mining Mita. *Econometrica*. 2010;78(6):1863-1903.

72. Huang W, Zhou Y. Effects of education on cognition at older ages: Evidence from China's Great Famine. *Soc Sci Med*. 2013;98:54-62.

73. Sotomayor O. Fetal and infant origins of diabetes and ill health: Evidence from Puerto Rico's 1928 and 1932 hurricanes. *Econ Hum Biol*. 2013;11(3):281-293.

74. Bhalotra S, Clots-Figueras I, Cassan G, Iyer L. Religion, politician identity and development outcomes: evidence from India. *J Econ Behav Organ.* 2014;104:4-17.

75. Zhong H. The effect of sibling size on children's health: a regression discontinuity design approach based on China's one-child policy. *China Econ Rev*. 2014;31:156-165.

76. Daysal NM, Trandafir M, van Ewijk R. Low-risk isn't no-risk: Perinatal treatments and the health of low-income newborns. *J Health Econ*. 2019;64:55-67.

77. Hutcheon JA, Harper S, Liauw J, Skoll MA, Srour M, Strumpf EC. Antenatal corticosteroid administration and early school age child development: A regression discontinuity study in British Columbia, Canada. *PLoS Med*. 2020;17(12):e1003435.

78. Brilli Y, Restrepo BJ. Birth weight, neonatal care, and infant mortality: Evidence from macrosomic babies. *Econ Hum Biol*. 2020;37:100825.

79. Bommer C, Horn S, Vollmer S. The effect of routine probiotics supplementation on preterm newborn health: a regression discontinuity analysis. *Am J Clin Nutr*. 2020;112(5):1219-1227.

80. Song D, Hurley JC, Lia M. Estimated treatment effects of tight glycaemic targets in mild gestational diabetes mellitus: a multiple cut-off regression discontinuity study design. Int J Environ Res Public Health. 2020;17(21):7725.

81. Harrison TM, Brown R, Duffey T, et al. Effects of massage on postoperative pain in infants with complex congenital heart disease. *Nurs Res*. 2020;69(5S):S36-S46.

82. Worsham CM, Woo J, Jena AB, Barnett ML. Adverse events and emergency department opioid prescriptions in adolescents. *Health Aff*. 2021;40(6):970-978.

83. Chyn E, Gold S, Hastings J. The returns to early-life interventions for very low birth weight children. *J Health Econ*. 2021;75:102400.

84. Holzhausen EA, Hagen EW, LeCaire T, Cadmus-Bertram L, Malecki KC, Peppard PE. A comparison of self- and proxy-reported subjective sleep durations with objective actigraphy measurements in a survey of Wisconsin children 6–17 years of age. *Am J Epidemiol*. 2021;190(5):755-765.

85. Furzer J, Dhuey E, Laporte A. ADHD misdiagnosis: Causes and mitigators. *Health Econ*. 2022;31(9):1926-1953.

86. Kim B, Thomsen MR, Nayga RM Jr, Fang D, Goudie A. Impact of Weight Status Reporting on Childhood Body Mass Index. Child Obes. 2022;18(7):485-493.

87. Hutcheon JA, Strumpf EC, Liauw J, et al. Antenatal corticosteroid administration and attention-deficit/hyperactivity disorder in childhood: a regression discontinuity study. *Can Med Assoc J*. 2022;194(7):E235-E241.

88. Tennant P, Doxford-Hook E, Flynn L, Kershaw K, Goddard J, Stacey T. Fasting plasma glucose, diagnosis of gestational diabetes and the risk of large for gestational age: a regression discontinuity analysis of routine data. *BJOG*. 2022;129(1):82-89.

89. van der Linde M, van Leeuwen N, Eijkenaar F, Rijneveld AW, Pieters R, Karim-Kos HE. Effect of treatment in a specialized pediatric hemato-oncology setting on 5-year survival in acute lymphoblastic leukemia: a quasi-experimental study. *Cancers (Basel)*. 2022;14(10):2451.

90. Nishioka D, Takaku R, Kondo N. Medical expenditure after marginal cut of cash benefit among public assistance recipients in Japan: natural experimental evidence. *J Epidemiol Community Health*. 2022;76(5):505-511.

91. Liu SY, Lim S. Difference in hospital utilization within the first 12 months among low-birth-weight infants in Medicaid managed care versus fee-for-service: a regression discontinuity study. *Matern Child Health J*. 2021;25(9):1410-1419.

92. Geiger CK, Clapp MA, Cohen JL. Association of prenatal care services, maternal morbidity, and perinatal mortality with the advanced maternal age cutoff of 35 years. *JAMA Health Forum*. 2021;2(12):e214044.

93. Epure AM, Courtin E, Wanner P, Chiolero A, Cullati S, Carmeli C. Effect of covering perinatal health-care costs on neonatal outcomes in Switzerland: a quasi-experimental population-based study. *Lancet Public Health*. 2023;8(3):e194-e202.

94. Fukuma S, Kato H, Takaku R, Tsugawa Y. Effect of no cost sharing for paediatric care on healthcare usage by household income levels: regression discontinuity design. *BMJ Open*. 2023;13(8):e071976.

95. Raifman J, Larson E, Barry CL, et al. State handgun purchase age minimums in the US and adolescent suicide rates: regression discontinuity and difference-in-differences analyses. BMJ. 2020;370:m2436.

96. de Chaisemartin C, de Chaisemartin L. Bacille Calmette-Guérin Vaccination in infancy does not protect against Coronavirus Disease 2019 (COVID-19): evidence from a natural experiment in Sweden. Clin Infect Dis. 2021;72(10):e501-e505.

97. Ahammer A, Bauernschuster S, Halla M, Lachenmaier H. Minimum legal drinking age and the social gradient in binge drinking. *J Health Econ*. 2022;81:102571.

98. Chuard C. Womb at work: The missing impact of maternal employment on newborn health. *J Health Econ*. 2020;73:102342.

99. Alfaro-Hudak KM, Schulkind L, Racine EF, Zillante A. SNAP and Cardiometabolic Risk in Youth. *Nutrients*. 2022;14(13):2756.

100. González L, Trommlerová S. Cash transfers before pregnancy and infant health. *J Health Econ*. 2022;83:102622.

101. Belenko S, Dennis M, Hiller M, et al. The impact of juvenile drug treatment courts on substance use, mental Health, and recidivism: results from a multisite experimental evaluation. *J Behav Health Serv Res*. 2022;49(4):436-455.

102. Proshin A. Impact of child subsidies on child health, well-being, and investment in child human capital: evidence from Russian Longitudinal Monitoring Survey 2010–2017. *Eur J Popul. 2023;39(1):14.*

103. Reader M. The infant health effects of starting universal child benefits in pregnancy: Evidence from England and Wales. *J Health Econ*. 2023;89:102751.

104. Bitler M, Currie J, Hoynes H, Ruffini K, Schulkind L, Willage B. Mothers as insurance: Family spillovers in WIC. *J Health Econ*. 2023;91:102784.

105. Rukiko MD, Mwakalobo ABS, Mmasa JJ. The impact of Conditional Cash Transfer program on stunting in under five year's poor children. Public Health Pract (Oxf). 2023;6:100437.

106. Johnson KM, Cheng L, Yin Y, et al. Impact of income-based public drug coverage deductibles on adherence to asthma medications. Ann Allergy Asthma Immunol. Published online October 21, 2023. doi:10.1016/j.anai.2023.10.017

107. Hong K, Dragan K, Glied S. Seeing and hearing: The impacts of New York City’s universal pre-kindergarten program on the health of low-income children. *J Health Econ*. 2019;64:93-107.

108. Courtin E, Nafilyan V, Avendano M, et al. Longer schooling but not better off? A quasi-experimental study of the effect of compulsory schooling on biomarkers in France. Soc Sci Med. 2019;220:379-386.

109. Plotnikov D, Williams C, Atan D, Davies NM, Ghorbani Mojarrad N, Guggenheim JA. Effect of education on myopia: evidence from the United Kingdom ROSLA 1972 reform. *Investig Opthalmol Vis Sci*. 2020;61(11):7.

110. Butler J, Black C, Craig P, et al. The long-term health effects of attending a selective school: a natural experiment. *BMC Med*. 2020;18(1):77.

111. He X, Sankaridurg P, Xiong S, et al. Prevalence of myopia and high myopia, and the association with education: Shanghai Child and Adolescent Large-scale Eye Study (SCALE): a cross-sectional study. *BMJ Open*. 2021;11(12):e048450.

112. Zhang C, Li L, Jan C, Li X, Qu J. Association of school education with eyesight among children and adolescents. *JAMA Netw Open*. 2022;5(4):e229545.

113. Muchomba FM. Effect of schooling on anemia and nutritional status among women: a natural experiment in Ethiopia. Am J Epidemiol. 2022;191(10):1722-1731.

114. Ye X, Zhu D, Ding R, He P. The effect of China’s compulsory education reforms on physiological health in adulthood: a natural experiment. *Health Policy Plan*. 2022;37(3):376-384.

115. Judd N, Sauce B, Klingberg T. Schooling substantially improves intelligence, but neither lessens nor widens the impacts of socioeconomics and genetics. *NPJ Sci Learn*. 2022;7(1):33.

116. Broughton T, Langley K, Tilling K, Collishaw S. Relative age in the school year and risk of mental health problems in childhood, adolescence and young adulthood. J Child Psychol Psychiatry. 2023;64(1):185-196.

117. Ding X, Morgan IG, Hu Y, et al. The causal effect of education on myopia: evidence that more exposure to schooling, rather than increased age, causes the onset of myopia. Invest Ophthalmol Vis Sci. 2023;64(4):25.

118. Gong J, Lu Y, Xie H. The average and distributional effects of teenage adversity on long-term health. *J Health Econ*. 2020;71:102288.

119. Fang Z, Chen C, Wang H, Tang K. Association Between Fetal Exposure to Famine and Anthropometric Measures in Adulthood: A Regression Discontinuity Approach. *Obesity*. 2020;28(5):962-969.

120. Aso S, Matsui H, Yasunaga H. Influence of the Fukushima Daiichi nuclear power plant accident on the use of computed tomography in children with mild head injuries. J Epidemiol. 2020;30(12):542-546.

121. Been J V, Burgos Ochoa L, Bertens LCM, Schoenmakers S, Steegers EAP, Reiss IKM. Impact of COVID-19 mitigation measures on the incidence of preterm birth: a national quasi-experimental study. *Lancet Public Health*. 2020;5(11):e604-e611.

122. Buitrago G, Moreno-Serra R. Conflict violence reduction and pregnancy outcomes: A regression discontinuity design in Colombia. *PLoS Med*. 2021;18(7):e1003684.

123. Bakolis I, Stewart R, Baldwin D, et al. Changes in daily mental health service use and mortality at the commencement and lifting of COVID-19 ‘lockdown’ policy in 10 UK sites: a regression discontinuity in time design. *BMJ Open*. 2021;11(5):e049721.

124. Takaku R, Yokoyama I. What the COVID-19 school closure left in its wake: Evidence from a regression discontinuity analysis in Japan. *J Public Econ*. 2021;195:104364.

125. Coma E, Català M, Méndez-Boo L, et al. Unravelling the role of the mandatory use of face covering masks for the control of SARS-CoV-2 in schools: a quasi-experimental study nested in a population-based cohort in Catalonia (Spain). *Arch Dis Child*. 2023;108(2):131-136.

126. Arif R, Chaudhry A, Chaudhry T. Empowered mothers and co-resident grandmothers: Two fundamental roles of women impacting child health outcomes in Punjab, Pakistan. *PLoS One*. 2023;18(11):e0285995.

127. Cattaneo MD, Jansson M, Ma X. Manipulation Testing Based on Density Discontinuity. *The Stata Journal: Promoting communications on statistics and Stata*. 2018;18(1):234-261. doi:10.1177/1536867X1801800115
